# Measuring neurodevelopment in iron-deficient children in sub-Saharan Africa: A systematic review of evidence and gaps

**DOI:** 10.1016/j.gloepi.2026.100274

**Published:** 2026-06-19

**Authors:** Maclean Vokhiwa, Nicola Pitchford, Lauren Cohee, Kamija S. Phiri, Eric Umar

**Affiliations:** a Kamuzu University of Health Sciences (KUHeS), Blantyre, Malawi; b Training and Research Unit of Excellence (TRUE), Zomba, Malawi; c International Centre for EdTech Impact, Stavanger, Norway; d Pediatrics and Child Health, Liverpool School of Tropical Medicine, Liverpool, UK

**Keywords:** Anemia, Iron deficiency, Neurodevelopment, Neuroimaging, Measurement equity, Africa

## Abstract

Anemia and iron-deficiency anemia are prevalent in sub-Saharan Africa (SSA) and linked to impaired brain and developmental outcomes. Progress in understanding these effects is constrained by inconsistent developmental measurement and minimal neuroimaging. This review evaluated how brain growth, cognition, and developmental status have been measured among children under five exposed to anemia, iron deficiency (ID), or iron-deficiency anemia (IDA) in SSA, to assess the availability, reliability, and limitations of neurodevelopmental measurement approaches and implications for comparability and interpretation.

A systematic review (PROSPERO CRD42024529208) was conducted following PRISMA guidelines. Six databases were searched for English-language studies published between Jan 1, 2013, and Apr 30, 2024, aligning with the WHO 2013–2025 global nutrition targets. Eligible studies included children aged 0–5 years in SSA exposed to anemia, ID, or IDA and reporting neurodevelopmental outcomes. Findings were synthesized narratively to characterize measurement heterogeneity, reliability reporting, and neuroimaging use. Evidence quality was appraised narratively based on study design and reporting completeness.

Of 2254 records screened, 19 studies from nine countries met inclusion criteria. Fourteen studies assessed developmental status, 13 assessed cognition, and only three measured brain outcomes using MRI or near-infrared spectroscopy. Substantial heterogeneity in measurement constructs, assessment tools, cultural adaptations, scoring approaches, and timing of assessment precluded quantitative meta-analysis. Reliability reporting was limited, with psychometric indices reported in seven of 14 developmental studies and nine of 13 cognitive studies. Only three studies incorporated direct measures of brain structure or function, limiting opportunities to relate behavioral outcomes to brain development.

Marked measurement heterogeneity, sparse reliability reporting, and the near-absence of neuroimaging constrain epidemiological inference, cross-study comparability, and mechanistic understanding of neurodevelopmental outcomes associated with anemia and ID in SSA. Strengthening culturally grounded assessment frameworks and more equitable, accessible approaches to objective brain measurement are priorities for improving measurement precision and understanding iron-related developmental outcomes.

## Research in context - panel

### Evidence before this study

Before initiating this review, existing evidence showed that anemia and iron deficiency are highly prevalent in sub-Saharan Africa and associated with developmental risks in early childhood. Numerous trials and cohort studies in the region have evaluated nutritional or micronutrient interventions, but the measurement of neurodevelopmental outcomes has been inconsistent and poorly documented. No prior synthesis had examined how developmental outcomes are measured, the extent to which tools are culturally adapted or validated, the availability, reliability, and limitations of neurodevelopmental measurement approaches, or whether available methods can capture the neurobiological pathways linking anemia to early brain development.

We searched PubMed, EMBASE, Web of Science, Scopus, Cochrane Library, and Wiley Online Library for English-language studies from Jan 1, 2013, to Apr 30, 2024, with supplementary grey-literature searches. We included quantitative studies conducted in sub-Saharan Africa that assessed children aged 0–5 years exposed to anemia, iron deficiency, or iron-deficiency anemia and reported at least one neurodevelopmental outcome. Nineteen studies met criteria. Designs, exposures, and outcomes were heterogeneous; psychometric reporting was limited; and only three studies incorporated any neuroimaging. Variation in constructs, cultural adaptation, measurement approaches, and assessment timing precluded meta-analysis.

### Added value of this study

This review is the first to systematically characterize neurodevelopmental measurement practices among children exposed to anemia, iron deficiency (ID), or iron-deficiency anemia (IDA) in sub-Saharan Africa across brain growth, cognition, and developmental status. It documents substantial between-study heterogeneity, infrequent reliability reporting, and minimal contextual validation of tools. Importantly, it highlights the near-total absence of neuroimaging – despite the centrality of brain pathways in understanding neurodevelopmental outcomes associated with anemia and iron deficiency – and connects these gaps to broader issues of measurement inequity, accessibility, and structural limitations within African health systems.

### Implications of all the available evidence

Current measurement practices are insufficient to generate precise, comparable evidence on how anemia and iron deficiency affect early neurodevelopment. Without standardized, culturally grounded assessment frameworks and more equitable and accessible approaches to objective brain measurement in settings where conventional neuroimaging remains unavailable, biological effects cannot be disentangled from methodological variability. Strengthening developmental measurement systems and creating conditions that support objective brain-based assessment are important priorities for improving mechanistic understanding, guiding nutrition and early-childhood policy, and ensuring that children in high-burden settings benefit from equitable and locally relevant evidence.

## Introduction

Anemia is a significant global health challenge affecting pregnant women and children under five years of age, particularly in less developed regions. According to global estimates, around 37% of pregnant women and 40% of children aged 6–59 months are anemic globally [1]. Anemia during pregnancy increases maternal risk of life-threatening complications during delivery or postpartum, while the foetus or infant faces risks of prematurity, low birth weight, and subsequently, infant anemia and impaired early development [2], [3]. Sub-Saharan Africa (SSA) carries a disproportionate share of this burden, hosting the second-largest population of stunted and underweight children. Prevention of anemia in both mothers and infants remains a major 2025 global nutrition target [4].

Anemia can result from multiple causes, including nutritional deficiencies, infections, and genetic conditions. Among these, iron-deficiency anemia (IDA) – caused by insufficient iron availability for hemoglobin synthesis – is globally the most common subtype [5]. In existing literature, studies differ in whether they report anemia broadly or specify IDA through biochemical measures such as ferritin or transferrin receptor concentrations. A smaller subset of studies assesses iron deficiency (ID) alone, referring to depleted iron stores that may or may not yet cause anemia [6]. These definitional differences across studies are important context for interpreting findings and reflect a broader measurement challenge in distinguishing between anemia of diverse origins and anemia directly attributable to iron deficiency.

**Fig. 1:**
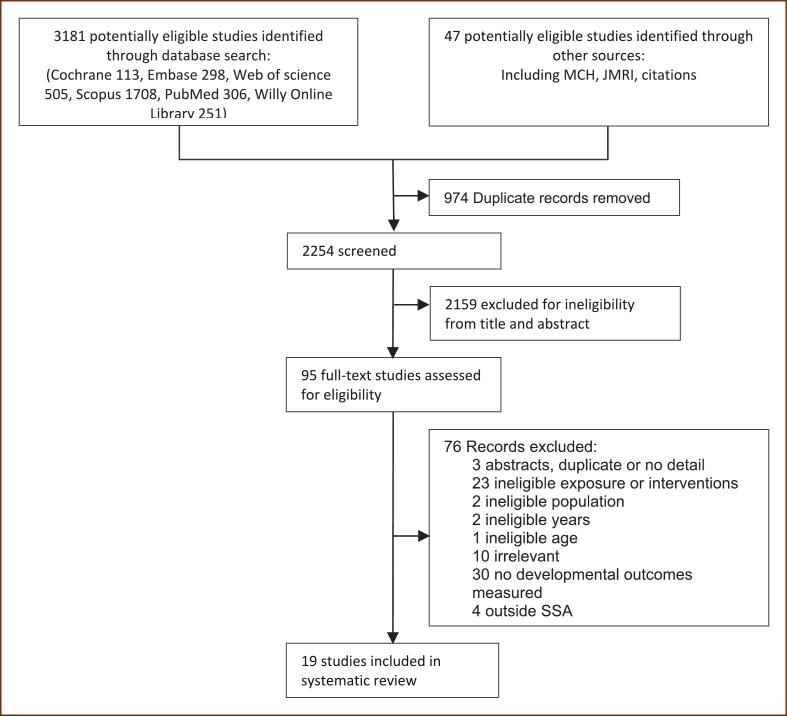
PRISMA flow diagram showing study selection process. Depicts the identification, screening, eligibility, and inclusion stages of the systematic review. Out of 2254 records identified through database searches, 2159 were excluded after title and abstract review. Ninety-five full-text articles were assessed for eligibility, and 19 studies met all inclusion criteria.

Iron plays multiple critical roles in the developing brain, including myelination of white matter, energy metabolism, and neurotransmitter synthesis, all of which are essential for fetal and early childhood brain growth [7], [8]. Iron deficiency during early development can produce impairments in cognitive and motor functions, some of which may persist despite later iron supplementation [9]. Iron status also influences the physical maturation of brain structures, which may be assessed using neuroimaging modalities that capture structural or functional aspects of brain development, including magnetic resonance imaging (MRI) [10]. MRI enables detailed, non-invasive examination of pediatric brain development [11]. Highlighted here as an example neuroimaging approach relevant to structural brain growth assessment, MRI remains largely inaccessible in low-resource regions because of high costs and technical demands [12]. The biological pathways for early neurodevelopment are shaped by multiple moderating factors – including maternal nutrition and infection, placental function, infant growth, socioeconomic context, and the quality of developmental or neuroimaging measurement tools – which may obscure associations and complicate cross-study comparisons. Given these pathways and influences (conceptually illustrated in Appendix 1, Fig. 1), accurate measurement of brain structure and developmental outcomes in iron-deficient populations remains a major methodological gap in SSA.

Understanding brain development among children exposed to anemia – and particularly iron-deficiency anemia – is essential for improving public-health outcomes, promoting socio-economic growth, and tracking progress toward the global health targets in SSA. Yet, the ability to quantify these developmental effects is limited by persistent measurement gaps [13]. The use of diverse measurement tools, varying constructs, differing assessment time-points, and limited neuroimaging access continues to hinder synthesis of evidence across studies in low-resource settings.

Against this background, this review systematically investigates how key neurodevelopmental outcomes – specifically brain growth, cognitive function, and developmental status (defined here as multidimensional developmental functioning and milestones across cognitive, language, motor, and socio-emotional domains) – have been measured among children under five years of age exposed to iron-deficiency or anemia in SSA. It further evaluates the availability and reliability of these measurements. By examining both between-study heterogeneity and within-study reliability, this review identifies critical gaps limiting cross-study comparison and understanding of neurodevelopmental outcomes associated with anemia and iron deficiency in early childhood. The review was undertaken with the expectation that existing studies would show substantial variability in measurement tools and limited reliability reporting, constraining comparability across contexts.

## Methods

### Search strategy and selection criteria

This systematic review followed PRISMA guidelines and was registered in PROSPERO (CRD42024529208). PubMed, EMBASE, Web of Science, Scopus, Cochrane Library, and Wiley Online Library were searched for English-language studies published from January 2013 to April 2024, supplemented by grey-literature searches of WHO reports, ClinicalTrials.gov, Google Scholar, and conference proceedings, alongside manual screening of reference lists from included studies and relevant reviews. The restriction to English-language publications was applied for feasibility and consistency of screening and data extraction, but may have reduced capture of relevant studies published in other languages used across SSA. The 2013 start date aligns with the WHO 2013–2025 global nutrition targets, which renewed global focus on childhood anemia and early development [4].

Eligible studies used quantitative designs (randomized or non-randomized trials, cohort, cross-sectional, or controlled before–after studies) and reported at least one postnatal neurodevelopmental outcome among children aged birth to 5 years in SSA. Studies were included if they assessed exposure to anemia, iron deficiency, or iron-deficiency anemia, or evaluated interventions addressing these conditions. Exposures could occur prenatally or postnatally, and outcomes measured at any point under age 5 were eligible. Studies conducted outside SSA or without relevant neurodevelopmental outcomes were excluded. Full search terms for one database are included in Appendix 2.

The primary outcomes were:

1. Brain growth (MRI or other neuroimaging measures)
2. Cognition (executive, memory, attentional, or general cognitive functioning)
3. Developmental status (motor, language, social, or adaptive functioning)
4. Measurement reliability (internal consistency, inter-observer agreement, or test–retest statistics)

Secondary outcomes included anemia/iron-deficiency prevalence, mean hemoglobin levels, and contextual developmental determinants.

### Study screening and data extraction

Two reviewers (TN, CK) independently screened titles and abstracts, followed by full-text review. Discrepancies were first discussed between reviewers and unresolved cases were adjudicated by the first author (MV) through review of the full text against the predefined eligibility criteria and study objectives specified in the review protocol.

Data were extracted into a standardized Excel form capturing study identifiers, design, setting, sample size, participant characteristics, exposure/intervention details, neurodevelopmental outcomes, measurement tools, and any available reliability statistics. Where studies indicated that additional methodological or psychometric information was available in supplementary materials, these materials were reviewed and incorporated into extraction. Study authors were not contacted to obtain unreported measurement reliability information. A full list of extracted variables appears in Appendix 3.

### Data synthesis

Given heterogeneity in study designs and outcomes, findings were synthesized narratively. Analyses focused on:

(1) the distribution of neurodevelopmental domains measured (brain growth, cognition, developmental status),
(2) the extent of reliability reporting, and.
(3) associations between iron-related exposures and neurodevelopmental outcomes.

Between-study measurement heterogeneity (variation in tools and constructs) was distinguished from within-study reliability reporting (measurement quality indicators). Secondary syntheses summarized anemia/iron-deficiency prevalence and developmental associations.

The overall quality of evidence was appraised narratively, focusing on study design, measurement approaches, completeness of reporting, and potential sources of bias, including variability in exposure definitions, outcome measurement, and reliability reporting.

Funders had no role in study design, data collection, data analysis, or manuscript preparation.

## Results

Nineteen studies met the inclusion criteria, revealing pronounced heterogeneity in outcome measurement, limited reliability reporting, and minimal use of neuroimaging approaches.

A total of 2254 records were identified from database searches and screened for eligibility. After exclusion of 2159 articles by title and abstract review, 95 full-text articles were assessed, of which 19 met inclusion criteria (Fig. 1; PRISMA flow diagram). List of studies excluded at full-text screening stage, with brief reasons is included in Appendix 4. The included studies were conducted across nine SSA countries – Uganda (4), Ghana (1), Malawi (3), South Africa (4), Ethiopia (2), Zimbabwe (1), Guinea-Bissau (2), Benin (1), and Niger (1).

Study designs included ten randomized controlled trials (including two with follow-up assessments), four hospital-based prospective cohorts and one community-based, one retrospective cohort, two cross-sectional studies, and one controlled pilot trial. Sample sizes ranged from 78 to 2186 participants, with a mean age of 23·9 months (range: 6–60 months). Developmental assessments were administered between 6 months and 5 years. Studies represented urban, peri-urban, and rural settings across both community and hospital recruitment, where hospital-based studies referred to participants recruited through healthcare services and community-based studies referred to participants recruited independent of illness presentation. Detailed characteristics are presented in Table 1.

**Table 1:** Characteristics of included studies (n = 19) conducted in Sub-Saharan Africa.

Author (Year)	Country	Design	Sample Size (% Female)	Mean Age (SD), Months / Assessment Time-Points	Exposure(s) / Intervention(s) / health	Outcomes Measured ⁎
Ssemata (2020a)	Uganda	RCT, hospital-community	239 (48.6%)	36.0 (0.9); baseline, 6, 12 mo FU	ID, daily oral ferrous sulfate (2 mg/kg/day); mean Hb 11.9 ± 0.2 g/dL	Cognition, Developmental status
Ocansey (2019)	Ghana	RCT, follow-up, hospital	966 (52.2%)	60.0 (0); 4–6 yr age	Maternal and infant malnutrition; LNS, MMN, IFAS; maternal Hb 11.2 ± 11.3 g/L	Cognition, Developmental status
Prado (2020)	Malawi	RCT, hospital-community	660 (47.7%)	7.4 (1.2); <12 mo age	Daily egg supplementation; anemia 60.9%	Cognition, Developmental status
Smuts (2019)	South Africa	RCT, community	750 (48.4%)	6.22 (0.25); 6, 12 mo age	Anemia; SQ-LNS, SQ-LNS-plus; Hb 117.7 g/L; anemia 23.7%, ID 23.4%, IDA 9.8%	Developmental status
Ssemata (2020b)	Uganda	Prospective cohort, hospital	259 (23.6%)	23.8 (0.95); 6–42 mo	Severe anemia	Developmental status
Ssemata (2020c)	Uganda	Prospective cohort, hospital	270 (23.6%)	23.8 (0.95); 6–42 mo	Severe anemia; blood transfusion treatment	Cognition
Rothman (2018)	South Africa	Cross-sectional, community	750 (48.4%)	6.0 (0); 6 mo	Anemia, stunting, ID; anemia 36.4%, ID 16.1%, IDA 10.5%	Developmental status
Nampijja (2022)	Uganda	Prospective cohort, community	993 (_%)	15.0; 15 mo, 5 yr	Anemia and ID; anemia 46.7% at 12 mo; ID 31.0% at 24 mo;	Cognition, Developmental status
Geletu (2019)	Ethiopia	Retrospective cohort, community	200 (50%)	10.7 (1.1); 9–12 mo age	Anemia; low-iron (6 mg) MNPs home fortification; Hb 11.8 ± 3.7 g/dL; anemia 41%	Developmental status
Mwangi (2023)	Malawi	RCT, hospital-community	2168 (_%)	12.0 (±14 days); 12, 18 mo	Anemia, malaria; iron syrup, MNP, malaria chemoprevention; 70% anemic (Hb <11 g/dL)	Cognition, Developmental status
Chandna (2020)	Zimbabwe	Cross-sectional in cluster RCT, community	323 (50%)	26.3 (2.1); 24 mo	HIV exposure; IYCF, WASH; small-quantity LNS; Hb diff 2.9 g/L (95% CI 0.9–4.9)	Cognition, Developmental status
Roberts (2017)	Guinea-Bissau	Controlled pilot trial, community	78 (_%)	36.0 (0); 11 wk–3 yr	Stunting, MAM; fortified supplement 291–350 kcal/day for 11 wk.; Hb 12.13 ± 0.82 g/dL	Brain, Cognition
Gashu (2016)	Ethiopia	RCT, community	541 (49.5%)	56.9 (1.8); 54–60 mo	Stunting, anemia; Se deficiency; anemia 13.6%, ID 9.1%, IDA 5.3%	Cognition, Developmental status
Roberts (2020)	Guinea-Bissau	RCT, community	1059 (46.4%)	33.6; 15–48 mo	Undernutrition; NEWSUP vs FBF; anemia 75.5%, Hb 9.9 (9.3–10.6)	Brain, Cognition
Wedderburn (2022)	South Africa	Prospective nested cohort, hospital	147 (43.5%)	34.0 (2.0); 2–3 yr	Maternal and child anemia; maternal 31.3%, child 52.5%	Brain
Rikhotso (2022)	South Africa	RCT, follow-up, community	392 (48.1%)	12.7 (0.52); 12, 18 mo	Stunting, poor nutrition; SQ-LNS (varied timing); Hb 118.6 ± 15.8; anemia 27.6%	Developmental status
Mireku (2016)	Benin	Prospective cohort, hospital	636 (_%)	12.0; 12 mo	Prenatal ID; maternal IPTp, anthelmintics, ferrous sulfate 200 mg + folic acid 5 mg daily	Cognition, Developmental status
Prado (2016)	Malawi	RCT, community	755 (53%)	18.4 (0.4); 6, 12, 18 mo	Maternal, infant undernutrition; SQ-LNS, IFA, MMN capsules daily	Cognition, Developmental status
Sudfeld (2022)	Niger	RCT, community	1632 (_%)	24.0; 6–24 mo	Poor maternal diet, anemia, micronutrient deficiencies; supplementation	Cognition, Developmental status

Notes: ID = iron deficiency; IDA = iron-deficiency anemia; Hb = hemoglobin; LNS = lipid-based nutrient supplement; SQ-LNS = small quantity lipid-based nutrient supplement; MMN = multiple micronutrient supplement; IFAS = iron and folic acid supplementation; IYCF = infant and young child feeding; WASH = water, sanitation, and hygiene; MNP = micronutrient powder; MAM = moderate acute malnutrition; NEWSUP = nutrient-enriched supplement; FBF = fortified blended food.

Studies are organized by author and year, summarizing design, country, sample characteristics, exposure type (anemia, ID, IDA, intervention, health status), and key neurodevelopmental outcome domains measured.

⁎ Assessment tools used are presented in Table 2 below.

Across studies, substantial heterogeneity existed in the selection, adaptation, and timing of neurodevelopmental measures (Table 2). Fourteen studies (74%) assessed developmental status, thirteen (68%) measured cognition, and only three (16%) incorporated direct measures of brain.

**Table 2:** Primary outcomes — neurodevelopmental outcomes, measures, and reliability.

Outcome Domain	n/N (%) Studies	Study	Construct(s)	Measurement Tool(s)	Reliability Reported
Brain growth	3/19 (16%)	Roberts (2017) [14]	Cerebral hemodynamics; cerebral blood flow index (CBFi)	Near-infrared spectroscopy (NIRS), Diffuse correlation spectroscopy (DCS)	–
		Roberts et al. (2020) [15]	Cerebral blood flow index (CBFi)	NIRS, DCS	–
		Wedderburn (2022) [10]	Brain volumes (global, subcortical, corpus callosum)	3 T Siemens Skyra MRI (T1-weighted)	QC for motion and segmentation errors
Cognition	13/19 (68%)	Ssemata (2020a) [19]	Cognitive learning, sustained attention, associative memory	MSEL, ECVT, COAT	–
		Ocansey (2019) [31]	Language, visuospatial, declarative memory, attention, inhibition, pre-academic skills	DNA-II, Block Design, PAL, Visual Search, DGID-EL, PEDS-p	*r* = 0.61–0.94; α = 0.61–0.9
		Prado (2020) [24]	Declarative memory, attention, imitation, IOWA task	Elicited Imitation Task, IOWA, VPC	ICC 0.90–0.92
		Ssemata (2020) [16]	Cognitive, language, motor z-scores	Bayley-III	–
		Nampijja (2022) [21]	Executive function (self-control, delay inhibition)	A-not-B Task	–
		Mwangi (2023) [17]	Cognitive composite score	Bayley-III	Inter-rater and 10% interobserver reliability
		Chandna (2020) [22]	Object permanence, cognition	A-not-B Test	ICC 0.85 (95% CI 0.80–0.90)
		Roberts (2017) [14]	Executive function, working memory	Spin the Pots Task	*r* > 0.9
		Gashu (2016) [25]	Fluid intelligence (verbal, nonverbal reasoning)	WPPSI-III subtests	–
		Roberts (2020) [15]	Executive function, working memory	Spin the Pots Task	r > 0.9
		Mireku (2016) [20]	Early Learning Composite	MSEL	–
		Prado (2016) [23]	Executive function – working memory	A-not-B Task	92% agreement; *r* = 0.75
		Sudfeld (2022) [18]	Cognitive function	Bayley-III	α ≥ 0.85
Developmental status	14/19 (74%)	Ssemata (2020) [27]	Behavioral problems, executive function, exploration, activity level	CBCL, BRIEF-P, BRS	BRS ICC > 0.8
		Ocansey (2019) [31]	Fine motor, social-emotional, psychosocial	NIH Pegboard, BRS, SDQ	α = 0.61–0.9
		Prado (2020) [24]	Motor, language, personal-social delay	MDAT	–
		Smuts (2019) [26]	Psychomotor, locomotor, motor milestones	Kilifi Developmental Inventory, SA Parent Scale, WHO milestones	–
		Ssemata (2020) [16]	Social-emotional, adaptive behavior	Bayley-III	–
		Rothman (2018) [28]	Psychomotor, locomotor, hand coordination	Kilifi Developmental Inventory, SA Parent Scale	–
		Nampijja (2022) [21]	Language, self-care, motor	Caregiver report, KDI	–
		Geletu (2019) [29]	Gross motor milestones	WHO Multicentre Growth Reference	–
		Mwangi (2023) [17]	Motor, language, behavior	Bayley-III, Wolke Behavior, Bates	10% interobserver testing
		Chandna (2020) [22]	Developmental status, motor, language, impulsivity	MDAT, CDI, Self-Control Task	ICC 0.88 (95% CI 0.82–0.94)
		Gashu (2016) [25]	Preliteracy, prenumeracy skills	School Readiness Test	–
		Rikhotso (2022) [30]	Psychomotor, eye–hand coordination	Kilifi Developmental Inventory, SA Parent Scale	–
		Prado (2016) [23]	Motor, language, social-emotional milestones	WHO Growth Study, MDAT, KDI, CDI, PSED	KDI 92%, PSED 80%, *r* = 0.65–0.82
		Sudfeld (2022) [18]	Language, motor, gross motor milestones	Bayley-III, WHO milestone report	α ≥ 0.85

Notes: MSEL = Mullen Scales of Early Learning; ECVT = Early Childhood Vigilance Test; COAT = Color Object Association Test; CBCL = Child Behavior Checklist; BRIEF-P = Behavior Rating Inventory of Executive Function (Preschool); BRS = Behavior Rating Scales; ICC = intraclass correlation coefficient; DNA-II = Developmental Neuropsychological Assessment II; DGID-EL = Delay of Gratification and International Development and Early Learning Assessment; SDQ = Strengths and Difficulties Questionnaire; PEDS-p = Parent's Evaluation of Developmental Status (pre-academic subscale).

Summarizes the measurement tools applied across brain growth, cognition, and developmental status domains, including details on cultural adaptation and psychometric reporting (e.g., Cronbach α, ICC).

*Brain growth*: Only three studies reported brain outcomes – two using near-infrared spectroscopy (NIRS) to assess cerebral blood-flow index (CBFi) [14], [15] and one using structural MRI to quantify global and regional brain volumes [10]. The MRI study implemented motion correction and segmentation quality control, demonstrating feasibility of pediatric neuroimaging in resource-constrained settings. The scarcity of neuroimaging approaches indicate a major measurement gap, although the limited number of studies in this review precludes broader conclusions regarding brain growth patterns across SSA populations.

*Cognition*: Cognitive outcomes were assessed using a diverse array of tools, including: Bayley Scales of Infant and Toddler Development, Third Edition (Bayley-III) [16], [17], [18], Mullen Scales of Early Learning (MSEL) [19], [20], A-not-B executive function task [21], [22], [23], Elicited Imitation Task, Infant Orienting With Attention (IOWA), and Visual Paired Comparison (VPC) [24], Wechsler Preschool and Primary Scale of Intelligence – Third Edition (WPPSI-III) subtests [25], Spin the Pots [14], [15], and other locally adapted tasks. Even when identical tools were used (e.g., Bayley-III), procedures differed considerably with respect to language translation, cultural adaptation, and scoring cut-offs.

*Developmental status*: Developmental assessments covered motor, language, social, behavioral, and adaptive skills, using instruments such as: Malawi Developmental Assessment Tool (MDAT), Kilifi Developmental Inventory (KDI), WHO motor milestones, Child Behavior Checklist (CBCL), Behavior Rating Inventory of Executive Function–Preschool Version (BRIEF-P), Strengths & Difficulties Questionnaire, MacArthur-Bates Communicative Development Inventories, and School Readiness Tests [26], [27], [28], [29], [30], [31]. Differences in domain emphasis, task format, and age of assessment (6–60 months) further contributed to heterogeneity.

As shown in Table 2, instruments differed substantially in the constructs and levels of neurodevelopment captured. Broad developmental tools such as Bayley-III and MSEL assessed multiple domains including cognition, language, and motor function, whereas the MDAT focused on gross motor, fine motor, language, and social development. Other approaches targeted narrower constructs, including executive function (A-not-B task), working memory (Spin the Pots), attention and recognition memory (Visual Paired Comparison), behavioral regulation (CBCL and BRIEF-P), communication (MacArthur-Bates Communicative Development Inventories), and school readiness. Outcome metrics also varied between standardized composite scores, milestone attainment, caregiver-reported outcomes, and direct task performance. Overall, this wide variation in constructs, instrument types, and assessment timing substantially limits cross-study comparability and meta-analytic synthesis.

Reliability reporting within studies was limited and patchy (Table 2). Among studies measuring developmental status, only seven of fourteen (50%) reported reliability indices; among cognition studies, nine of thirteen (69%) provided any reliability evidence. Reported reliability statistics included:

• Intraclass correlation coefficients (ICC: 0·80–0·92)
• Correlations (*r* = 0·61–09·4)
• Internal consistency (Cronbach α = 0·61–0·90)
• Assessor agreement rates (93–97%).

Although several tools were adapted linguistically (e.g., Chichewa, Kiswahili, Luganda), very few studies reported formal psychometric validation for local contexts, while others applied western or other prior norms directly. For instance, while a study in Malawi reported local validation of the MDAT, KDI, and Parent's Evaluation of Developmental Status (PSED) [23], some studies in Uganda and Benin did not report local cultural adaptation, re-norming, or psychometric validation of the Mullen Scales of Early Learning (MSEL) [19], [20]. Bayley-III was used without reporting locally normative references [16]. Limited reliability reporting reduces confidence in within-study estimates and heightens the risk of systematic measurement bias.

**Fig. 2:**
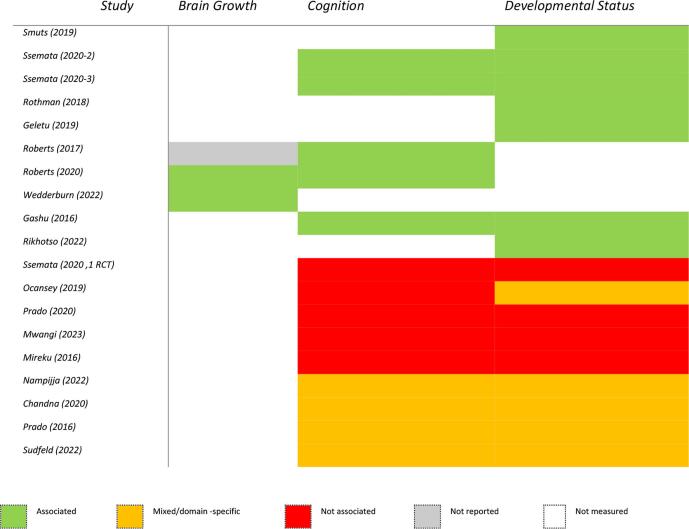
Matrix of associations between iron-status exposures and child neurodevelopmental outcomes (n = 19 studies) across Sub-Saharan Africa. Color shading indicates the direction and consistency of reported associations for each outcome domain. The visualization highlights substantial heterogeneity in measurement focus and findings across studies, underscoring the need for standardized and accessible methods – particularly low-field MRI – to advance equitable neurodevelopmental research in low-resource settings.

Findings on the relationship between anemia, ID, IDA, and neurodevelopment varied widely across the evidence base (Table 3). Across the nineteen included studies, ten (53%) reported associations between iron-related exposures or interventions and variation in neurodevelopmental outcomes, five (26%) reported no clear association, and four (21%) reported mixed findings (see Fig. 2). The narrative examples below illustrate representative patterns; full details for all studies are provided in Table 3.

**Table 3:** Secondary outcome findings – associations.

Association Category (n/N, %)	Study (Author, Year, Country)	Sample / Design	Main Exposure(s)	Outcome Domain(s)	Key Findings	Effect estimate (Including *p*-values where reported)
Associated (10/19, 53%)	Smuts et al., 2019, South Africa	RCT (*n* = 750, community)	Anemia; SQ-LNS vs SQ-LNS-plus	Motor, Developmental	SQ-LNS improved locomotor development and parent-rated development scores	Mean difference: 2.05 (95% CI: 0.72–3.38), *p* = 0.003; Mean difference: 1.10 (95% CI: 0.14–2.07), *p* = 0.03
	Ssemata et al., 2020, Uganda (2)	Prospective cohort (*n* = 259)	Severe anemia	Cognitive, Socio-emotional	Severe anemia was associated with lower socio-emotional and cognitive outcomes.	β = −0.29 (SE 0.05), *p* = 0.002
	Ssemata et al., 2020, Uganda (3)	Cohort (*n* = 270)	Severe anemia	Cognition, Language, Motor	Anemia was associated with lower cognitive, language, and motor outcomes.	β = −0.20, *p* = 0.02; β = −0.25, *p* < 0.0001; β = −0.17, *p* = 0.05
	Rothman et al., 2018, South Africa	Cross-sectional (n = 750)	Anemia, Stunting	Psychomotor	Psychomotor performance was associated with child growth status	*p* < 0.01
	Geletu et al., 2019, Ethiopia	Retrospective cohort (*n* = 200)	Low-iron MNP	Motor	Low-iron MNP improved motor milestone achievement	AOR = 3.1, p < 0.0001
	Roberts et al., 2017, Guinea-Bissau	Pilot trial (*n* = 78)	Nutrient supplementation	Cognition	Higher hemoglobin was associated with better working memory and task-switching	*p* < 0.05
	Roberts et al., 2020, Guinea-Bissau	RCT (*n* = 1059)	NEWSUP vs FBF	Cognition, Brain	Supplementation improved working memory and cerebral blood flow.	p = 0.03; *p* = 0.04
	Wedderburn et al., 2022, South Africa	Nested cohort (*n* = 147)	Maternal anemia	Brain volume	Maternal anemia was associated with reduced brain volumes	Caudate −5.3%; Putamen −4.3%; Corpus callosum −7.8%
	Gashu et al., 2016, Ethiopia	Community RCT (*n* = 541)	Anemia, Stunting	Cognition	Anemia was associated with lower reasoning and school readiness	p < 0.01
	Rikhotso et al., 2022, South Africa	RCT (*n* = 392)	Delayed SQ-LNS	Motor	Delayed SQ-LNS was associated with improved eye–hand coordination	*p* = 0.09
Not associated (5/19, 26%)	Ssemata et al., 2020, Uganda (1)	RCT (*n* = 239)	Iron therapy	Cognition, Behavior	No clear differences in cognition or behavior between groups	*p* ≥ 0.06
	Ocansey et al., 2019, Ghana	RCT (*n* = 966)	LNS, MMN, IFAS	Cognition, Socio-emotional	No clear differences in outcomes; some improvement in social-emotional behavior.	*p* = 0.01
	Prado et al., 2020, Malawi	RCT (*n* = 660)	Egg diet	Cognition, Development	No clear differences across developmental domains	Not reported
	Mwangi et al., 2023, Malawi	RCT (*n* = 1944)	Iron syrup, MNP, chemoprevention	Cognition, Language, Motor	No differences across cognitive, language, and motor outcomes	Not reported
	Mireku et al., 2016, Benin	Cohort (*n* = 636)	Prenatal iron deficiency	Cognition, Motor	No association between prenatal iron deficiency and developmental outcomes	Not reported
Mixed (4/19, 21%)	Nampijja et al., 2022, Uganda	Prospective cohort (*n* = 993)	Anemia in pregnancy, infant Hb	Psychomotor, Language	Associations observed at 15 months but not at 5 years	Not reported
	Chandna et al., 2020, Zimbabwe	Cluster RCT (*n* = 323)	IYCF, WASH, LNS	Cognition, Language	Improved vocabulary and developmental scores; no effect on fine motor outcomes	Not reported
	Prado et al., 2016, Malawi	RCT (*n* = 755)	LNS, IFA, MMN	Motor, Language	Variable effects across domains and subgroups	Not reported
	Sudfeld et al., 2022, Niger	RCT (*n* = 1632)	Prenatal LNS vs IFA	Cognition, Language, Motor	No differences in mean scores; improved early milestone attainment	HR = 1.57, p = 0.01

Notes: LNS = lipid-based nutrient supplements; MMN = multiple micronutrients; IFA = iron–folic acid; MNP = micronutrient powders.

Summarizes direction and magnitude of associations between anemia, iron deficiency (ID), and neurodevelopmental outcomes (brain growth, cognition, language, motor, socio-emotional, and developmental status) across studies conducted in sub-Saharan Africa.

Several studies reported associations between improved iron status and better neurodevelopmental outcomes:

• Smuts 2019 (South Africa): lipid-based nutrient supplementation improved hemoglobin levels and locomotor development [26].
• Ssemata 2020 (Uganda; a, b): severe anemia was associated with lower cognitive, language, and motor scores [19], [27].
• Roberts 2017, 2020 (Guinea-Bissau): higher hemoglobin was associated with better task-switching and working memory performance, and supplementation improved working memory and cerebral blood-flow index [14], [15].
• Wedderburn 2022 (South Africa): maternal anemia was associated with reduced caudate, putamen, and corpus callosum volumes on MRI [10].
• Rothman 2018 (South Africa): psychomotor development was associated with child hemoglobin and length-for-age z-scores [28].
• Gashu 2016 (Ethiopia): reasoning and school readiness scores were lower among children with anemia or stunting [25].
• Geletu 2019 (Ethiopia): low-iron micronutrient powder supplementation improved achievement of motor milestones [29].
• Rikhotso 2022 (South Africa): delayed small quantity lipid-based nutrient supplements (SQ-LNS) supplementation was associated with modest improvements in eye–hand coordination [30].

Five studies found no clear associations between iron status or interventions and neurodevelopmental outcomes:

• Ssemata 2020 (Uganda, c): no clear differences in cognitive or behavioral outcomes at 12-month follow-up after immediate vs delayed iron therapy [16].
• Ocansey 2019 (Ghana): no group differences in cognition or developmental status across supplementation arms [31].
• Prado 2020 (Malawi): no clear differences in cognitive or developmental outcomes following a daily egg diet intervention [24].
• Mwangi 2023 (Malawi): no effects of iron syrup, multiple micronutrient powders, or chemoprevention on Bayley-III scores [17].
• Mireku 2016 (Benin): prenatal iron deficiency was not associated with cognitive or motor outcomes at 12 months [20].

Four studies reported domain-specific or time-specific effects:

• Nampijja 2022 (Uganda): anemia in pregnancy and low infant hemoglobin were associated with lower language and psychomotor scores at 15 months, but not at 5 years [21].
• Chandna 2020 (Zimbabwe): vocabulary and general development improved, but fine motor and object permanence did not [22].
• Prado 2016 (Malawi): supplementation showed early advantages in selected motor milestones, but effects were not consistent across domains or time [23].
• Sudfeld 2022 (Niger): no differences in Bayley-III scores, but prenatal LNS increased the likelihood of achieving certain early gross-motor milestones [18].

These divergent findings likely reflect possible biological variability – including differences in timing of exposure (prenatal versus postnatal), severity of anemia or iron deficiency, developmental stage at assessment, and coexisting nutritional or infectious conditions – and substantial differences in measurement tools, cultural adaptations, and timing of developmental assessments.

Across studies, overall evidence quality varied. Among interventional and observational studies, common limitations included heterogeneous measurement approaches, inconsistent reporting of psychometric properties, variability in exposure definitions, and differences in study design. These factors introduce potential sources of bias and limit comparability across studies.

## Discussion

This systematic review identified substantial variability in how neurodevelopmental outcomes are measured among children under five exposed to anemia and iron deficiency across SSA. While anemia, ID, and IDA remain highly prevalent in the region [1], [5], these burdens persist within a broader context of inequities in neonatal and child health, where improvements in aggregate health indicators may obscure unequal access to resources, infrastructure, and opportunities for generating contextually relevant evidence in low-resource settings [32]. Within this context, there was minimal use of neuroimaging to quantify brain growth – only three of the nineteen studies incorporated any form of imaging. This limited use of neuroimaging highlights constrained capacity to directly evaluate the biological pathways through which anemia and iron deficiency affect child neurodevelopment. Understanding these pathways requires both behavioral and brain-based developmental measures; however, most studies relied solely on observational or psychometric tools that were variably adapted and sparsely validated [33], [34].

A balanced measurement framework should treat behavioral assessments and neuroimaging as complementary. Behavioral and caregiver-reported developmental tools are often more feasible, lower cost, scalable, and culturally adaptable in resource-limited settings, while remaining essential for assessing functional developmental outcomes. However, their interpretation depends on local adaptation, assessor training, language equivalence, and psychometric validation [35], [36], [37]. Neuroimaging can add mechanistic information on brain structure, growth, and function that may not be captured by behavioral tools alone, but its use is constrained by cost, infrastructure, technical expertise, motion sensitivity, and limited availability across SSA [38], [39], [40], [41], [42]. A central challenge is the limited integration of culturally grounded developmental assessments with feasible neuroimaging approaches.

Consistent with this, across included studies, measurement heterogeneity emerged as the most consistent challenge [33], [34], [43]. The studies used diverse tools - often developed in high-income contexts - to evaluate similar constructs such as cognition, motor development, or socio-emotional behavior [20], [21], [22], [23], [24],. Even when the same instruments were used, they were applied at different ages, administered in different languages, or variably adapted to cultural settings. For example, adaptations of the Bayley Scales differed across Malawi, Ghana, and Uganda, where one study did not report local normative adjustments or psychometric validation [16], [17], [18]. The continued reliance on developmental tools originally developed and normed outside African contexts likely reflects broader structural challenges rather than methodological preference alone. Local validation, re-norming, translation equivalence testing, and assessor standardization require sustained funding, psychometric expertise, and sufficiently large reference populations [33]. These requirements may be difficult to secure in low-resource settings where researchers may rely on internationally recognised instruments because they facilitate comparison across studies and are more familiar to funders, journals, and collaborative research networks. Together, these structural and methodological factors complicate synthesis and limits the comparability of developmental outcomes across studies and populations [43], [44], [45].

Furthermore, the use of different tools, constructs, scoring systems, exposure definitions, and assessment ages precluded quantification of the specific contribution of each methodological difference to the synthesis of cognitive and developmental outcomes. Instead, the review identifies likely pathways through which measurement heterogeneity may influence interpretation: broad composite tools may dilute domain-specific effects, culturally unvalidated tools may introduce construct nonequivalence, and differences in timing of assessment may obscure developmental windows during which exposures, such as anemia or iron deficiency, have stronger associations with neurodevelopment [43]. Interpretation of these patterns should also consider that the review was restricted to English-language publications and may therefore underrepresent evidence published in other languages across SSA. As a result, the observed distribution of developmental measurement approaches and regional research gaps may not fully reflect the broader neurodevelopment literature across the region.

Recent initiatives are beginning to address these challenges through the development of standardized developmental assessment approaches intended for use across diverse settings, including low- and middle-income countries. For example, emerging frameworks such as the Global Scales for Early Development (GSED) aim to improve comparability while retaining contextual relevance across populations [36], [37]. Although such initiatives represent important progress toward harmonized developmental measurement, implementation at scale may still require investment in local validation infrastructure, normative data generation, and long-term psychometric capacity. In addition, these approaches do not fully address the limited integration of direct brain-based measures identified in this review [38], [41]. Together with limited reliability reporting and sparse integration of neuroimaging, these measurement gaps constrain interpretation of neurodevelopmental outcomes and limit mechanistic understanding of how anemia and iron deficiency influence early brain development.

Limited and patchy reporting of assessment reliability within studies constrained confidence in reported developmental and cognitive outcomes [33], [34]. Only about half of the studies assessing developmental outcomes and two-thirds of those measuring cognition reported reliability statistics, such as internal consistency or interrater agreement. Where reported, values generally indicated acceptable psychometric strength (Cronbach's α = 0·61–0·90; ICC = 0·80–0·92). However, the absence of validation for adapted tools undermines confidence in these estimates, as some assessments were translated or abbreviated without re-testing [43]. This gap introduces potential measurement bias and weakens both internal validity and cross-cultural comparability [32], [44], [45].

The lack of standardized measurement frameworks further constrains interpretation of associations between anemia or iron deficiency and neurodevelopmental outcomes. While approximately half of the included studies reported associations – showing that maternal or infant iron supplementation improved hemoglobin levels and developmental performance - others reported no clear or mixed patterns of association [10], [21], [24]. These inconsistencies likely reflect variation in measurement precision, timing of assessment, and contextual factors such as infection, nutrition, and socioeconomic status [8], [9], [43]. Without standardized tools, it remains difficult to disentangle biological effects from methodological differences [43], [46].

The limited use of neuroimaging compounds these challenges. Only one study used MRI to quantify brain volume - growth, while two applied near-infrared spectroscopy to measure cerebral blood flow [10], [14], [15]. These examples demonstrate the potential value of brain-based measurement and the systemic barriers limiting wider implementation, including high costs, limited infrastructure, inconsistent electricity, and a shortage of trained personnel in SSA [12], [47], [48]. Successful neuroimaging initiatives in SSA further demonstrate both the opportunities and practical demands of this approach. For example, the Brain Imaging for Global Health (BRIGHT) study in The Gambia has used portable neuroimaging, including fNIRS, alongside developmental and behavioral assessments to characterize early brain and cognitive development, including analyses of iron status and later developmental trajectories [41], [42], [49]. Such work shows that longitudinal brain-based measurement can be conducted in African settings when supported by strong local partnerships, infrastructure planning, and context-appropriate protocols [41], [42]. Neuroimaging may provide additional value alongside behavioral assessment by detecting structural and functional brain alterations that are not always captured through developmental testing alone, potentially offering mechanistic insight into how early anemia and iron deficiency influence neurodevelopment [38]. However, interpretation should remain cautious, as only three studies in this review incorporated direct brain measures and the available evidence base remains insufficient to support broad inference regarding neurodevelopmental trajectories in SSA. Integrating accessible imaging approaches alongside standardized behavioral tools may therefore represent one pathway toward reducing this measurement divide where contextually appropriate [39], [40]. Broader progress, however, will depend not only on imaging technologies themselves but also on strengthening measurement ecosystems that support equitable, accessible, and locally relevant approaches to understanding child neurodevelopment.

This review had several strengths, including the comprehensive inclusion of diverse study designs, mapping of neurodevelopmental measurement tools across sub-Saharan Africa, and focused appraisal of reliability reporting – an often-overlooked aspect of pediatric research [50]. However, the review was limited by the small number of studies incorporating neuroimaging, substantial heterogeneity in developmental measures, patchy reporting of reliability indices, and restriction to English-language publications, which may have reduced representation of evidence published in other languages across SSA. Together, these factors constrained comparability and synthesis across studies. Consequently, this review could not isolate the independent contribution of specific tools or cultural adaptations to observed differences across studies and instead provides a narrative synthesis of how methodological heterogeneity may influence interpretation. These findings align with previous reviews identifying methodological heterogeneity as a central barrier to pooled estimation and cumulative inference [46]. Overall, the strength of evidence varied across studies, reflecting differences in study design, measurement consistency, and completeness of reporting.

In conclusion, this review highlights three interconnected measurement gaps: (1) substantial heterogeneity in developmental tools across studies, (2) limited within-study reliability reporting, and (3) minimal integration of neuroimaging to directly quantify brain growth. Together, these limitations constrain current understanding and provide insufficient evidence to characterize neurodevelopmental effects at population level. Addressing these gaps will require strengthening culturally grounded developmental measurement while creating conditions that support more equitable and accessible approaches to objective brain measurement in settings where conventional neuroimaging remains unavailable. Such efforts are essential to improve measurement precision, strengthen causal inference, and advance equitable understanding of iron-related brain and developmental outcomes.

Future progress in this field depends on regional investment in standardized, culturally grounded assessment frameworks, alongside dedicated support for local tool validation, psychometric strengthening, and context-specific normative data generation. Where direct assessment of brain development is considered important, future work should prioritise approaches that improve accessibility and reduce measurement inequities while remaining responsive to local infrastructure, workforce, and financing realities. Rather than assuming expansion of conventional neuroimaging capacity, there is a need to explore context-appropriate pathways for integrating objective brain-based measures within broader child-development research ecosystems. This may include low-field or portable MRI systems that can be easily deployed in low-resource settings. Experience from initiatives such as BRIGHT suggests that feasibility depends not only on device portability, but also on sustained investment in local capacity, data quality systems, community engagement, and cross-disciplinary collaboration [41], [42]. Such approaches have the potential to transform how early brain growth and cognitive outcomes are quantified, fostering equitable advances in child neurodevelopment research, neuroscience, policy, and clinical care across Africa [40], [47], [51].

## CRediT authorship contribution statement

**Maclean Vokhiwa:** Writing – review & editing, Writing – original draft, Project administration, Methodology, Investigation, Formal analysis, Data curation, Conceptualization. **Nicola Pitchford:** Writing – review & editing, Supervision. **Lauren Cohee:** Writing – review & editing, Supervision. **Kamija S. Phiri:** Writing – review & editing, Supervision, Methodology, Conceptualization. **Eric Umar:** Writing – review & editing, Validation, Supervision, Methodology, Conceptualization.

## Funding

10.13039/100010269Wellcome Trust Grant Number(s): DEL-22-002, Science for Africa Foundation (DELTAS Africa); Gates Foundation Grant Number(s): INV-010612 (Training and Research Unit of Excellence - TRUE, maternal anemia trial, Malawi), and INV-090982 (Ultra-Low-Field Neuroimaging in the Young -UNITY).

## Declaration of competing interest

We declare no competing interests.

## Data Availability

Data collected for this systematic review – including the full PubMed search strategy (Appendix 2), the list of excluded full-text studies with reasons (Appendix 4), the deidentified study-level dataset, coding framework, and data dictionary – will be made available upon publication of this Article and will remain accessible indefinitely. The study protocol and data extraction form will also be accessible. Data can be requested from the corresponding author (mvokhiwa@gmail.com) and will be shared after review of a brief analysis proposal and completion of a data access agreement. All materials will be provided in deidentified form and made available without investigator support unless clarification is required.
