# Supplementary material for: Measuring neurodevelopment in iron-deficient children in sub-Saharan Africa: A systematic review of evidence and gaps
Source: Glob Epidemiol. 2026 Jun 19;12:100274. doi: 10.1016/j.gloepi.2026.100274 (PMC13320419; doi:10.1016/j.gloepi.2026.100274)
Supplement: Supplementary file 1 — Supplementary material [file mmc1.docx]

**SUPPLEMETARY MATERIALS**

Title

Measuring Neurodevelopment in Iron-Deficient Children in Sub-Saharan Africa: A Systematic Review of Evidence and Gaps

Running Title

Neurodevelopment Measurement Gaps in SSA

Subtitle

A synthesis of measurement heterogeneity, reliability reporting, and neuroimaging gaps

Authors

Maclean Vokhiwa (MPH)^1,2^, Nicola Pitchford (PHD)^3^, Lauren Cohee (PHD)^4^, Kamija S. Phiri (PHD)^2^, Eric Umar (PHD)^1^

Affiliations

1. Kamuzu University of Health Sciences (KUHeS), Blantyre, Malawi

2. Training and Research Unit of Excellence (TRUE), Zomba, Malawi

3. International Centre for EdTech Impact, Stavanger, Norway

4. Pediatrics and Child Health, Liverpool School of Tropical Medicine, Liverpool, UK

Corresponding Author

Maclean Vokhiwa, MPH

Kamuzu University of Health Sciences (KUHeS), Private Bag 360 Chichiri, Blantyre 3, Malawi

Email: [mvokhiwa@gmail.com](mailto:mvokhiwa@gmail.com)

Phone: +265999080096

Table of Contents

[Appendix 1: Figure conceptually illustrating biological pathways linking iron status to early neurodevelopment 3](#_Toc231287413)

[Appendix 2: Full search terms for databases 4](#_Toc231287414)

[PUBMED Search Strategy 4](#_Toc231287415)

[Embase Search Strategy 4](#_Toc231287416)

[Web of Science Search Strategy 5](#_Toc231287417)

[Scopus Search Strategy 6](#_Toc231287418)

[Cochrane Search Strategy 6](#_Toc231287419)

[Wiley Online Library Search Strategy 8](#_Toc231287420)

[Appendix 3: List of extracted variables and Study Eligibility form 9](#_Toc231287421)

[List of extracted variables 9](#_Toc231287422)

[Study eligibility form 10](#_Toc231287423)

[Appendix 4: List of studies excluded at full-text screening stage, with brief reasons. 11](#_Toc231287424)

[Appendix 5. PRISMA Checklist 15](#_Toc231287425)

# Appendix 1: Figure conceptually illustrating biological pathways *linking iron status to early neurodevelopment*

**Exposures**

**Outcomes**

Maternal iron

Placental Function

Fetal brain development

Infant iron

Brain growth

Cognition

Developmental status / milestones/ achievement

**Factors of influence at time of assessment**: child characteristics (age, sex, prematurity, stunting, head circumference, etc.), environment (stimulation, caregiving, preschool attendance, adversity, etc.), socioeconomic or contextual (maternal education, SES, test language, culture, examiner characteristics, etc.), measurement-related (translations, cultural equivalence, reliability, familiarity, fatigue, etc.), etc.

**Factors of influence**: biological or health (malnutrition, infections, inflammation, gestational age, breastfeeding, etc.), dietary or environmental (iron intake, hygiene conditions, toxin exposure, etc.) socioeconomic (maternal education, access to antenatal care and supplementation, etc.), etc.

Structure, activity

Measures: quantitative, e.g., MRI, EEG

Attention, memory, executive functions, etc.

Measures: individually-administered assessments, e.g., Bayley,

Language, motor, social, learning

Measure: parent/guardian reports, direct observation, e.g., MDAT, Bayley scales, school readiness tests, etc.

ID, anemia, hemoglobin levels, etc.

Measures: quantitative, e.g., lab tests

Placental health, infection, malaria, etc.

Measures: quantitative, e.g., lab tests

Structure, characteristics, etc.

Measures: quantitative, e.g., ultrasound, MRI

*Figure 1: Conceptual pathways linking iron status to early neurodevelopment.*

The diagram illustrates key biological mechanisms and the roles of maternal, placental, and infant factors in shaping observed neurodevelopmental outcomes, alongside contextual and measurement-related factors that may influence these pathways.

# Appendix 2: Full search terms for databases

## PUBMED Search Strategy

("africa south of the sahara"[MeSH Terms] OR "africa south of the sahara"[All Fields] OR "Sub-Saharan Africa"[Title/Abstract] OR "Subsahara"[Title/Abstract] OR "Central Africa"[Title/Abstract] OR "middle Africa"[Title/Abstract] OR "east africa"[Title/Abstract] OR "South Africa"[Title/Abstract] OR "west africa"[Title/Abstract] OR "Angola"[Title/Abstract] OR "Benin"[Title/Abstract] OR "Botswana"[Title/Abstract] OR "Burkina Faso"[Title/Abstract] OR "Burundi"[Title/Abstract] OR "Cameroon"[Title/Abstract] OR "Cape Verde"[Title/Abstract] OR "Central African Republic"[Title/Abstract] OR "Chad"[Title/Abstract] OR "Comoros"[Title/Abstract] OR "Congo"[Title/Abstract] OR "Cote d'Ivoire"[Title/Abstract] OR "Djibouti"[Title/Abstract] OR "Equatorial Guinea"[Title/Abstract] OR "Eritrea"[Title/Abstract] OR "Ethiopia"[Title/Abstract] OR "Gabon"[Title/Abstract] OR "Gambia"[Title/Abstract] OR "Ghana"[Title/Abstract] OR "Guinea"[Title/Abstract] OR "Guinea-Bissau"[Title/Abstract] OR "Kenya"[Title/Abstract] OR "Lesotho"[Title/Abstract] OR "Liberia"[Title/Abstract] OR "Madagascar"[Title/Abstract] OR "Malawi"[Title/Abstract] OR "Mali"[Title/Abstract] OR "Mauritania"[Title/Abstract] OR "Mauritius"[Title/Abstract] OR "Mozambique"[Title/Abstract] OR "Namibia"[Title/Abstract] OR "Niger"[Title/Abstract] OR "Nigeria"[Title/Abstract] OR "Reunion"[Title/Abstract] OR "Rwanda"[Title/Abstract] OR "Sao Tome and Principe"[Title/Abstract] OR "Senegal"[Title/Abstract] OR "Seychelles"[Title/Abstract] OR "Sierra Leone"[Title/Abstract] OR "Somalia"[Title/Abstract] OR "South Africa"[Title/Abstract] OR "Sudan"[Title/Abstract] OR "Swaziland"[Title/Abstract] OR "Eswatini"[Title/Abstract] OR "Tanzania"[Title/Abstract] OR "Togo"[Title/Abstract] OR "Uganda"[Title/Abstract] OR "Western Sahara"[Title/Abstract] OR "Zambia"[Title/Abstract] OR "Zimbabwe"[Title/Abstract]) AND (((("Brain"[MeSH Terms] OR "Brain"[Title/Abstract] OR "brain imaging"[Title/Abstract] OR "brain growth"[Title/Abstract] OR "brain development"[Title/Abstract] OR "brain structure"[Title/Abstract] OR "brain volume*"[Title/Abstract] OR "white matter"[Title/Abstract] OR "gray matter"[Title/Abstract] OR "cerebrospinal fluid"[Title/Abstract] OR "volumetric*"[Title/Abstract] OR "brain mri"[Title/Abstract] OR "Neuroimaging"[MeSH Terms] OR "Neuroimaging"[Title/Abstract] OR "mri"[Title/Abstract] OR "magnetic resonance imaging"[Title/Abstract] OR "magnetic resonance"[Title/Abstract] OR "eeg"[Title/Abstract] OR "electroencephalography"[Title/Abstract] OR "neurodevelopment*"[Title/Abstract] OR "neur*"[Title/Abstract]) OR ("Cognition"[MeSH Terms] OR "cogniti*"[Title/Abstract] OR "cognitive function"[Title/Abstract])) AND ("Learning"[MeSH Terms] OR "learn*"[Title/Abstract] OR "learning difficult*"[Title/Abstract] OR "SEN"[Title/Abstract] OR "SEND"[Title/Abstract] OR "special educational needs"[Title/Abstract] OR "learning disabilit*"[Title/Abstract] OR "Learning Disabilities"[MeSH Terms] OR "learning disorder*"[Title/Abstract] OR "attention deficit hyperactivity disorder*"[Title/Abstract] OR "ADHD"[Title/Abstract] OR "Attention Deficit Disorder with Hyperactivity"[MeSH Terms] OR "Autism Spectrum Disorder"[MeSH Terms] OR "autism spectrum disorder*"[Title/Abstract] OR "autis*"[Title/Abstract] OR "dyslexia"[Title/Abstract] OR "Memory"[Title/Abstract] OR "Memory"[MeSH Terms] OR "mental illness*"[Title/Abstract] OR "depression"[Title/Abstract] OR "anxiety"[Title/Abstract] OR "Behavior"[Title/Abstract] OR "Behavior"[MeSH Terms] OR "emotion"[Title/Abstract] OR "Emotions"[MeSH Terms] OR "disabilit*"[Title/Abstract] OR "handicap"[Title/Abstract] OR "chronic illness"[Title/Abstract] OR "Chronic Disease"[MeSH Terms] OR "developmental delay"[Title/Abstract] OR "Developmental Disabilities"[MeSH Terms] OR "developmental status"[Title/Abstract] OR "child development"[MeSH Terms] OR "child development*"[Title/Abstract] OR "neurodisabilit*"[Title/Abstract] OR "gross motor"[Title/Abstract] OR "fine motor"[Title/Abstract] OR "language"[Title/Abstract] OR "social abilit*"[Title/Abstract])) AND (("child*"[Title/Abstract] OR "child, preschool"[MeSH Terms] OR "Infant"[MeSH Terms] OR "Infant"[Title/Abstract] OR "paediatric"[Title/Abstract] OR "pediatric"[Title/Abstract] OR "Pediatrics"[MeSH Terms] OR "early years"[Title/Abstract] OR "preschool"[Title/Abstract]) AND ("Anaemia"[Title/Abstract] OR "anemia"[Title/Abstract] OR "iron deficiency"[Title/Abstract] OR "iron"[Title/Abstract] OR "malnutrition"[Title/Abstract] OR "nutrition*"[Title/Abstract] OR "nutritional status"[MeSH Terms] OR "hemoglobin"[Title/Abstract] OR "haemoglobin"[Title/Abstract] OR "Hb"[Title/Abstract] OR "maternal anemia"[Title/Abstract] OR "nutrient*"[Title/Abstract] OR "maternal health"[Title/Abstract] OR "child health"[Title/Abstract] OR "malnourished"[Title/Abstract] OR "malarial anemia"[Title/Abstract] OR "plasmodi*"[Title/Abstract] OR "falciparum"[Title/Abstract] OR "malaria"[MeSH Terms] OR "malaria"[Title/Abstract])))

Filters: **Humans, English, Infant: birth-23 months, Infant: 1-23 months, Preschool Child: 2-5 years, from 2013 – 2024**

**Date of final search: April 30, 2024, Time coverage: Jan 1, 2013 – Apr 30, 2024**

## Embase Search Strategy

Brain growth

#1

'brain'/exp OR 'brain':ti,ab,kw OR 'brain imaging':ti,ab,kw OR 'brain growth':ti,ab,kw OR 'brain development':ti,ab,kw OR 'brain structure':ti,ab,kw OR 'brain volume*':ti,ab,kw OR 'white matter':ti,ab,kw OR 'gray matter':ti,ab,kw OR 'cerebrospinal fluid':ti,ab,kw OR 'volumetric*':ti,ab,kw OR 'brain mri':ti,ab,kw OR 'neuroimaging'/exp OR 'neuroimaging':ti,ab,kw OR 'mri':ti,ab,kw OR 'magnetic resonance imaging':ti,ab,kw OR 'magnetic resonance':ti,ab,kw OR 'eeg':ti,ab,kw OR 'electroencephalography':ti,ab,kw OR 'neurodevelopment*':ti,ab,kw OR 'neur*':ti,ab,kw

OR

cognition

#2

'cognition'/exp OR 'cogniti*':ti,ab,kw OR 'cognitive function':ti,ab,kw

Brain growth OR cognition

#3 = #1 OR #2

#1 OR #2

AND

Learning abilities

#4

'learning'/exp OR 'learn*':ti,ab,kw OR 'learning difficult*':ti,ab,kw OR 'sen':ti,ab,kw OR 'send':ti,ab,kw OR 'special educational needs':ti,ab,kw OR 'learning disabilit*':ti,ab,kw OR 'learning disorder'/exp OR 'learning disorder*':ti,ab,kw OR 'attention deficit hyperactivity disorder*':ti,ab,kw OR 'adhd':ti,ab,kw OR 'attention deficit hyperactivity disorder'/exp OR 'autism'/exp OR 'autism spectrum disorder*':ti,ab,kw OR 'autis*':ti,ab,kw OR 'dyslexia':ti,ab,kw OR 'memory':ti,ab,kw OR 'memory'/exp OR 'mental illness*':ti,ab,kw OR 'depression':ti,ab,kw OR 'anxiety':ti,ab,kw OR 'behavior':ti,ab,kw OR 'behavior'/exp OR 'emotion':ti,ab,kw OR 'emotion'/exp OR 'disabilit*':ti,ab,kw OR 'handicap':ti,ab,kw OR 'chronic illness':ti,ab,kw OR 'chronic disease'/exp OR 'developmental delay':ti,ab,kw OR 'developmental disorder'/exp OR 'developmental status':ti,ab,kw OR 'child development'/exp OR 'child development*':ti,ab,kw OR 'neurodisabilit*':ti,ab,kw OR 'gross motor':ti,ab,kw OR 'fine motor':ti,ab,kw OR 'language':ti,ab,kw OR 'social abilit*':ti,ab,kw

AND

Early years Children

#5

'child*':ti,ab,kw OR 'preschool child'/exp OR 'infant'/exp OR 'infant':ti,ab,kw OR 'paediatric':ti,ab,kw OR 'pediatric':ti,ab,kw OR 'pediatrics'/exp OR 'early years':ti,ab,kw OR 'preschool':ti,ab,kw

AND

Anaemia

#6

'anaemia':ti,ab,kw OR 'anemia':ti,ab,kw OR 'iron deficiency':ti,ab,kw OR 'iron':ti,ab,kw OR 'malnutrition':ti,ab,kw OR 'nutrition*':ti,ab,kw OR 'nutritional status'/exp OR 'hemoglobin':ti,ab,kw OR 'haemoglobin':ti,ab,kw OR 'hb':ti,ab,kw OR 'maternal anemia':ti,ab,kw OR 'nutrient*':ti,ab,kw OR 'maternal health':ti,ab,kw OR 'child health':ti,ab,kw OR 'malnourished':ti,ab,kw OR 'malarial anemia':ti,ab,kw OR 'plasmodi*':ti,ab,kw OR 'falciparum':ti,ab,kw OR 'malaria'/exp OR 'malaria':ti,ab,kw

AND

Sub-Saharan Africa

#7

'Africa south of the Sahara'/exp OR 'africa south of the sahara' OR 'sub-saharan africa':ti,ab,kw OR 'subsahara':ti,ab,kw OR 'central africa':ti,ab,kw OR 'middle africa':ti,ab,kw OR 'east africa':ti,ab,kw OR 'south africa':ti,ab,kw OR 'west africa':ti,ab,kw OR 'angola':ti,ab,kw OR 'benin':ti,ab,kw OR 'botswana':ti,ab,kw OR 'burkina faso':ti,ab,kw OR 'burundi':ti,ab,kw OR 'cameroon':ti,ab,kw OR 'cape verde':ti,ab,kw OR 'central african republic':ti,ab,kw OR 'chad':ti,ab,kw OR 'comoros':ti,ab,kw OR 'congo':ti,ab,kw OR 'cote d`ivoire':ti,ab,kw OR 'djibouti':ti,ab,kw OR 'equatorial guinea':ti,ab,kw OR 'eritrea':ti,ab,kw OR 'ethiopia':ti,ab,kw OR 'gabon':ti,ab,kw OR 'gambia':ti,ab,kw OR 'ghana':ti,ab,kw OR 'guinea':ti,ab,kw OR 'guinea-bissau':ti,ab,kw OR 'kenya':ti,ab,kw OR 'lesotho':ti,ab,kw OR 'liberia':ti,ab,kw OR 'madagascar':ti,ab,kw OR 'malawi':ti,ab,kw OR 'mali':ti,ab,kw OR 'mauritania':ti,ab,kw OR 'mauritius':ti,ab,kw OR 'mozambique':ti,ab,kw OR 'namibia':ti,ab,kw OR 'niger':ti,ab,kw OR 'nigeria':ti,ab,kw OR 'reunion':ti,ab,kw OR 'rwanda':ti,ab,kw OR 'sao tome' AND 'principe':ti,ab,kw OR 'senegal':ti,ab,kw OR 'seychelles':ti,ab,kw OR 'sierra leone':ti,ab,kw OR 'somalia':ti,ab,kw OR 'south africa':ti,ab,kw OR 'sudan':ti,ab,kw OR 'swaziland':ti,ab,kw OR 'eswatini':ti,ab,kw OR 'tanzania':ti,ab,kw OR 'togo':ti,ab,kw OR 'uganda':ti,ab,kw OR 'western sahara':ti,ab,kw OR 'zambia':ti,ab,kw OR 'zimbabwe':ti,ab,kw

(Brain growth OR cognition) AND Learning

#8 = #3 AND #4

Early years Children AND anaemia

#9 = #5 AND #6

[(Brain growth OR cognition) AND Learning Abiities]

AND

[Early Years Children AND anaemia]

#10 = #8 AND #9

{[(Brain growth OR cognition) AND Learning Abilities]

AND

[Early Years Children AND anaemia]}

AND

Sub-Saharan Africa

#11 = #7 AND #10

**Filters**: …AND #7 AND #10 AND [2013-2024]/py AND [humans]/lim AND [english]/lim AND ([infant]/lim OR [preschool]/lim) AND ([embase]/lim NOT ([embase]/lim AND [medline]/lim) OR [preprint]/lim)

**Date of final search: April 30, 2024, Time coverage: Jan 1, 2013 – Apr 30, 2024**

## Web of Science Search Strategy

Brain growth

#1

TS=("Brain" OR "brain imaging" OR "brain growth" OR "brain development" OR "brain structure" OR "brain volume*" OR "white matter" OR "gray matter" OR "cerebrospinal fluid" OR "volumetric*" OR "brain mri" OR "Neuroimaging" OR "mri" OR "magnetic resonance imaging" OR "magnetic resonance" OR "eeg" OR "electroencephalography" OR "neurodevelopment*" OR "neur*")

OR

cognition

#2

TS=("Cognition" OR "cogniti*" OR "cognitive function")

Brain growth OR cognition

#3 = #1 OR #2

AND

Learning abilities

#4

(TS=("Learning" OR "learn*" OR "learning difficult*" OR "SEN" OR "SEND" OR "special educational needs" OR "learning disabilit*" OR "Learning Disabilities" OR "learning disorder*" OR "attention deficit hyperactivity disorder*" OR "ADHD" OR "Attention Deficit Disorder with Hyperactivity" OR "autism spectrum disorder*" OR "autis*" OR "dyslexia" OR "Memory" OR "mental illness*" OR "depression" OR "anxiety" OR "Behavior" OR "emotion" OR "disabilit*" OR "handicap" OR "chronic illness" OR "Chronic Disease" OR "developmental delay" OR "Developmental Disabilities" OR "developmental status" OR "child development" OR "neurodisabilit*" OR "gross motor" OR "fine motor" OR "language" OR "social abilit*"))

AND

Early years Children

#5

TS=("child*" OR "child, preschool" OR "Infant" OR "paediatric" OR "pediatric" OR "Pediatrics" OR "early years" OR "preschool")

AND

Anaemia

#6

TS=("Anaemia" OR "anemia" OR "iron deficiency" OR "iron" OR "malnutrition" OR "nutrition*" OR "nutritional status" OR "hemoglobin" OR "haemoglobin" OR "Hb" OR "maternal anemia" OR "nutrient*" OR "maternal health" OR "child health" OR "malnourished" OR "malarial anemia" OR "plasmodi*" OR "falciparum" OR "malaria" OR "malaria")

AND

Sub-Saharan Africa

#7

TS=("africa south of the sahara" OR “Sub-Saharan Africa” OR “Sub sahara” OR “Central Africa” OR “middle Africa” OR “East* Africa” OR “South* Africa” OR “West* Africa” OR "Angola" OR "Benin" OR "Botswana" OR “Burkina Faso” OR "Burundi" OR "Cameroon" OR “Cape Verde” OR “Central African Republic” OR "Chad" OR "Comoros" OR "Congo" OR “Côte d'Ivoire” OR "Djibouti" OR “Equatorial Guinea” OR "Eritrea" OR "Ethiopia" OR "Gabon" OR "Gambia" OR "Ghana" OR "Guinea" OR “Guinea-Bissau” OR "Kenya" OR "Lesotho" OR "Liberia" OR "Madagascar" OR "Malawi" OR "Mali" OR "Mauritania" OR "Mauritius" OR "Mozambique" OR "Namibia" OR "Niger" OR "Nigeria" OR "Réunion" OR "Rwanda" OR “Sao Tome " OR "Principe" OR "Senegal" OR "Seychelles" OR “Sierra Leone” OR "Somalia" OR “South Africa” OR "Sudan" OR "Swaziland" OR "Eswatini" OR "Tanzania" OR "Togo" OR "Uganda" OR “Western Sahara” OR "Zambia" OR "Zimbabwe")

Combinations:

(Brain growth OR cognition) AND Learning Abilities

#8 = #3 AND #4

Early Years Children AND Anaemia

#9 = #5 AND #6

[(Brain growth OR cognition) AND Learning Abilities]

AND

[Early Years Children AND Anaemia]

#10 = #8 AND #9

{[(Brain growth OR cognition) AND Learning Abilities]

AND

[ Early Years Children AND anaemia]}

AND

Sub-Saharan Africa

#11

#7 AND #10

**Filters**: #7 AND #10 and 2024 or 2023 or 2022 or 2021 or 2020 or 2019 or 2018 or 2017 or 2016 or 2015 or 2014 or 2013 (Publication Years) and Humans (MeSH Headings) and English (Languages) and Child or Adolescent or Child Preschool or Infant (MeSH Headings) and ANGOLA or BENIN or BOTSWANA or BURKINA FASO or BURUNDI or CAMEROON or CENT AFR REPUBL or CHAD or COTE IVOIRE or DEM REP CONGO or DEMOCRATIC REPUBLIC OF CONGO or DJIBOUTI or EQUAT GUINEA or ERITREA or ETHIOPIA or ESWATINI or GABON or GAMBIA or GHANA or GUINEA or GUINEA BISSAU or KENYA or LESOTHO or LIBERIA or MADAGASCAR or MALAWI or MALI or MAURITANIA or MAURITIUS or MOZAMBIQUE or MOCAMBIQUE or NIGER or NAMIBIA or NIGERIA or REP CONGO or RWANDA or SAO TOME PRIN or SENEGAL or SEYCHELLES or SIERRA LEONE or SOMALIA or SOUTH AFRICA or SOUTH SUDAN or SUDAN or SWAZILAND or TANZANIA or TOGO or UGANDA or ZAMBIA or ZIMBABWE (Countries/Regions)

**Date of final search: April 30, 2024, Time coverage: Jan 1, 2013 – Apr 30, 2024**

## Scopus Search Strategy

( ( ( ( TITLE-ABS-KEY ( brain OR ( brain AND imaging ) OR ( brain AND growth ) OR ( brain AND development ) OR ( brain AND structure ) OR ( brain AND volume* ) OR ( white AND matter ) OR ( gray AND matter ) OR ( cerebrospinal AND fluid ) OR volumetric* OR ( brain AND mri ) OR neuroimaging OR mri OR ( magnetic AND resonance AND imaging ) OR ( magnetic AND resonance ) OR eeg OR electroencephalography OR neurodevelopment* OR neur* ) ) OR ( TITLE-ABS-KEY ( cognition OR cogniti* OR ( cognitive AND function ) ) ) ) AND ( TITLE-ABS-KEY ( learning OR learn* OR ( learning AND difficult* ) OR sen OR send OR ( special AND educational AND needs ) OR ( learning AND disabilit* ) OR ( learning AND disabilities ) OR ( learning AND disorder* ) OR ( attention AND deficit AND hyperactivity AND disorder* ) OR adhd OR ( attention AND deficit AND disorder AND with AND hyperactivity ) OR ( autism AND spectrum AND disorder* ) OR autis* OR dyslexia OR memory OR ( mental AND illness* ) OR depression OR anxiety OR behavior OR emotion OR disabilit* OR handicap OR ( chronic AND illness ) OR ( chronic AND disease ) OR ( developmental AND delay ) OR ( developmental AND disabilities ) OR ( developmental AND status ) OR ( child AND development* ) OR neurodisabilit* OR ( gross AND motor ) OR ( fine AND motor ) OR language OR ( social AND abilit* ) ) ) ) AND ( ( TITLE-ABS-KEY ( child* OR ( child, AND preschool ) OR infant OR paediatric OR pediatric OR pediatrics OR ( early AND years ) OR preschool ) ) AND ( TITLE-ABS-KEY ( anaemia OR anemia OR ( iron AND deficiency ) OR iron OR malnutrition OR nutrition* OR ( nutritional AND status ) OR hemoglobin OR haemoglobin OR hb OR ( maternal AND anemia ) OR nutrient* OR ( maternal AND health ) OR ( child AND health ) OR malnourished OR ( malarial AND anemia ) OR plasmodi* OR falciparum OR malaria ) ) ) ) AND ( TITLE-ABS-KEY ( ( africa AND south AND sahara ) OR ( sub-saharan AND africa ) OR subsahara OR ( central AND africa ) OR ( middle AND africa ) OR ( east* AND africa ) OR ( south* AND africa ) OR ( west* AND africa ) OR angola OR benin OR botswana OR ( burkina AND faso ) OR "burkina faso" OR burundi OR cameroon OR ( cape AND verde ) OR "cape verde" OR ( central AND african AND republic ) OR "central african republic" OR chad OR comoros OR congo OR ( côte AND d'ivoire ) OR "côte d'ivoire" OR djibouti OR ( equatorial AND guinea ) OR "equatorial guinea" OR eritrea OR ethiopia OR gabon OR gambia OR ghana OR guinea OR ( guinea-bissau ) OR "guinea-bissau" OR kenya OR lesotho OR liberia OR madagascar OR malawi OR mali OR mauritania OR mauritius OR mozambique OR namibia OR niger OR nigeria OR réunion OR rwanda OR ( sao AND tome AND principe ) OR "sao tome and principe" OR ( sao AND tome ) OR senegal OR seychelles OR ( sierra AND leone ) OR "sierra leone" OR somalia OR ( south AND africa ) OR "south africa" OR sudan OR swaziland OR eswatini OR tanzania OR togo OR uganda OR ( western AND sahara ) OR "western sahara" OR zambia OR zimbabwe ) )

Filters: PUBYEAR > 2012 AND PUBYEAR < 2025, humans, English, country limit, age

**Date of final search: April 30, 2024, Time coverage: Jan 1, 2013 – Apr 30, 2024**

## Cochrane Search Strategy

**Brain growth**

**#1**

**("Brain"):ti,ab,kw OR ("brain imaging"):ti,ab,kw OR ("brain growth"):ti,ab,kw OR ("brain development"):ti,ab,kw OR ("brain structure"):ti,ab,kw OR ("brain volume") OR ("white matter"):ti,ab,kw OR ("gray matter"):ti,ab,kw OR ("cerebrospinal fluid"):ti,ab,kw OR ("volumetric"):ti,ab,kw OR ("brain mri"):ti,ab,kw OR ("Neuroimaging"):ti,ab,kw OR ("mri"):ti,ab,kw OR ("magnetic resonance imaging"):ti,ab,kw OR ("magnetic resonance"):ti,ab,kw OR ("eeg"):ti,ab,kw OR ("electroencephalography"):ti,ab,kw OR ("neurodevelopment"):ti,ab,kw OR( "neur"):ti,ab,kw**

**#2**

**MeSH descriptor: [Brain] explode all trees**

**#3**

**MeSH descriptor: [Neuroimaging] explode all trees**

**#4 = #1 OR #2 OR #3**

**OR**

**cognition**

**#5**

**(“cogniti”):ti,ab,kw OR (“cognitive function”):ti,ab,kw**

**#6**

**MeSH descriptor: [Cognition] explode all trees**

**#7 = #5 OR #6**

**Brain growth OR cognition**

**#8 = #4 OR #7**

**AND**

**Learning Abilities**

**#9**

**("learn"):ti,ab,kw OR ("learning difficult"):ti,ab,kw OR ("SEN"):ti,ab,kw OR ("SEND"):ti,ab,kw OR ("special educational needs"):ti,ab,kw OR ("learning disabilit"):ti,ab,kw OR ("learning disorder"):ti,ab,kw OR ("attention deficit hyperactivity disorder"):ti,ab,kw OR ("ADHD"):ti,ab,kw OR ("autism spectrum disorder"):ti,ab,kw OR ("autis"):ti,ab,kw OR ("dyslexia"):ti,ab,kw OR ("Memory"):ti,ab,kw OR ("mental illness"):ti,ab,kw OR ("depression"):ti,ab,kw OR ("anxiety"):ti,ab,kw OR ("Behavior"):ti,ab,kw OR ("emotion"):ti,ab,kw OR ("disabilit"):ti,ab,kw OR ("handicap"):ti,ab,kw OR ("chronic illness"):ti,ab,kw OR ("developmental delay"):ti,ab,kw OR ("developmental status"):ti,ab,kw OR ("child development"):ti,ab,kw OR ("neurodisabilit"):ti,ab,kw OR ("gross motor"):ti,ab,kw OR ("fine motor"):ti,ab,kw OR ("language"):ti,ab,kw OR ("social abilit"):ti,ab,kw**

**#10**

**MeSH descriptor: [Learning] explode all trees**

**#11**

**MeSH descriptor: [Learning Disabilities] explode all trees**

**#12**

**MeSH descriptor: [Attention Deficit Disorder with Hyperactivity] explode all trees**

**#13**

**MeSH descriptor: [Autism Spectrum Disorder] explode all trees**

**#14**

**MeSH descriptor: [Memory] explode all trees**

**#15**

**MeSH descriptor: [Behavior] explode all trees**

**#16**

**MeSH descriptor: [Emotions] explode all trees**

**#17**

**MeSH descriptor: [Chronic Disease] explode all trees**

**#18**

**MeSH descriptor: [Developmental Disabilities] explode all trees**

**#19**

**MeSH descriptor: [Child Development] explode all trees**

**#20 = #9 OR #10 OR #11 OR #12 OR #13 OR #14 OR #15 OR #16 OR #17 OR #18 OR #19**

**AND**

**Early Years Children**

**#21**

**(“child”):ti,ab,kw OR (“infant”):ti,ab,kw OR (“paediatric”):ti,ab,kw OR (“pediatric”):ti,ab,kw OR (“early years”):ti,ab,kw OR (“preschool”):ti,ab,kw**

**#22**

**MeSH descriptor: [Child, Preschool] explode all trees**

**#23**

**MeSH descriptor: [Infant] explode all trees**

**#24**

**MeSH descriptor: [Pediatrics] explode all trees**

**#25 = #21 OR #22 OR #23 OR #24**

**AND**

**Anaemia**

**#26**

**("Anaemia"):ti,ab,kw OR ("anemia"):ti,ab,kw OR ("iron deficiency"):ti,ab,kw OR ("iron"):ti,ab,kw OR ("malnutrition"):ti,ab,kw OR ("nutrition"):ti,ab,kw OR ("hemoglobin"):ti,ab,kw OR ("haemoglobin"):ti,ab,kw OR ("Hb"):ti,ab,kw OR ("maternal anemia"):ti,ab,kw OR ("nutrient"):ti,ab,kw OR ("maternal health"):ti,ab,kw OR ("child health"):ti,ab,kw OR ("malnourished"):ti,ab,kw OR ("malarial anemia"):ti,ab,kw OR ("plasmodi"):ti,ab,kw OR ("falciparum"):ti,ab,kw OR ("malaria"):ti,ab,kw**

**#27**

**MeSH descriptor: [Nutritional Status] explode all trees**

**#28**

**MeSH descriptor: [Malaria] explode all trees**

**#29 = #26 OR #27 OR #28**

**AND**

**Sub-Saharan Africa**

**#30**

**(africa south of the sahara) OR (“Sub-Saharan Africa”):ti,ab,kw OR (“Subsahara”):ti,ab,kw OR (“Central Africa”):ti,ab,kw OR (“middle Africa”):ti,ab,kw OR (“East Africa”):ti,ab,kw OR (“Eastern Africa”):ti,ab,kw OR (“South Africa”):ti,ab,kw OR (“Southern Africa”):ti,ab,kw OR (“West Africa”):ti,ab,kw OR (“Western Africa”):ti,ab,kw OR (“Angola”):ti,ab,kw OR (“Benin”):ti,ab,kw OR (“Botswana”):ti,ab,kw OR (“Burkina Faso”):ti,ab,kw OR (“Burundi”):ti,ab,kw OR (“Cameroon”):ti,ab,kw OR (“Cape Verde”):ti,ab,kw OR (“Central African Republic”):ti,ab,kw OR (“Chad”):ti,ab,kw OR (“Comoros”):ti,ab,kw OR (“Congo”):ti,ab,kw OR (“Côte d'Ivoire”):ti,ab,kw OR (“Djibouti”):ti,ab,kw OR (“Equatorial Guinea”):ti,ab,kw OR (“Eritrea”):ti,ab,kw OR (“Ethiopia”):ti,ab,kw OR (“Gabon”):ti,ab,kw OR (“Gambia”):ti,ab,kw OR (“Ghana”):ti,ab,kw OR (“Guinea”):ti,ab,kw OR (“Guinea-Bissau”):ti,ab,kw OR (“Kenya”):ti,ab,kw OR (“Lesotho”):ti,ab,kw OR (“Liberia”):ti,ab,kw OR (“Madagascar”):ti,ab,kw OR (“Malawi”):ti,ab,kw OR (“Mali”):ti,ab,kw OR (“Mauritania”):ti,ab,kw OR (“Mauritius”):ti,ab,kw OR (“Mozambique”):ti,ab,kw OR (“Namibia”):ti,ab,kw OR (“Niger”):ti,ab,kw OR (“Nigeria”):ti,ab,kw OR (“Réunion”):ti,ab,kw OR (“Rwanda”):ti,ab,kw OR (“Sao Tome and Principe”):ti,ab,kw OR (“Sao Tome”):ti,ab,kw OR (“Principe”):ti,ab,kw OR (“Senegal”):ti,ab,kw OR (“Seychelles”):ti,ab,kw OR (“Sierra Leone”):ti,ab,kw OR (“Somalia”):ti,ab,kw OR (“South Africa”):ti,ab,kw OR (“Sudan”):ti,ab,kw OR (“Swaziland”):ti,ab,kw OR (“Eswatini”):ti,ab,kw OR (“Tanzania”):ti,ab,kw OR (“Togo”):ti,ab,kw OR (“Uganda”):ti,ab,kw OR (“Western Sahara”):ti,ab,kw OR (“Zambia”):ti,ab,kw OR (“Zimbabwe”):ti,ab,kw**

**#31**

**MeSH descriptor: [Africa South of the Sahara] explode all trees**

**#32 = #30 OR #31**

**(Brain growth OR cognition) AND Learning Abilities**

**#33 = #8 AND #20**

**Early Years Children AND Anaemia**

**#34 = #25 AND #29**

**[(Brain growth OR cognition) AND Learning Abilities]**

**AND**

**[Early Years Children AND anaemia]**

**#35 = #33 AND #34**

**{[(Brain growth OR cognition) AND Learning Abilities]**

**AND**

**[Early Years Children AND anaemia]}**

**AND**

**Sub-Saharan Africa**

**#36 = #30 AND #35**

**Filters: Humans, English, Child: birth-18 years, Infant: birth-23 months, Infant: 1-23 months, Newborn: birth-1 month, Preschool Child: 2-5 years, Child: 6-12 years, Adolescent: 13-18 years, from 2013 – 2024**

**Date of final search: April 30, 2024, Time coverage: Jan 1, 2013 – Apr 30, 2024**

## Wiley Online Library Search Strategy

**"Brain OR "brain imaging" OR "brain growth" OR "brain development" OR "brain structure" OR "brain volume" OR "white matter" OR "gray matter" OR "grey matter" OR "cerebrospinal fluid" OR volumetric* OR "brain mri" OR neuroimaging OR mri OR "magnetic resonance imaging" OR "magnetic resonance" OR eeg OR electroencephalography OR neurodevelopment* OR neur* OR cogniti* OR "cognitive function"" anywhere and "learn* OR "learning difficult" OR sen OR send OR "special educational needs" OR "learning disabilit" OR "learning disorder" OR "attention deficit hyperactivity disorder" OR adhd OR "autism spectrum disorder" OR autis* OR dyslexia OR memory OR "mental illness" OR depression OR anxiety OR behavior OR emotion OR disabilit OR handicap OR "chronic illness" OR "Chronic Disease" OR "developmental delay" OR "developmental disabilities" OR "developmental status" OR "child development" OR neurodisabilit* OR "gross motor" OR "fine motor" OR "language" OR "social ability"" anywhere and "child* OR Infant OR p*diatric OR "early years" OR preschool" anywhere and "an*mia OR "iron deficiency" OR iron OR malnutrition OR nutrition* OR "nutritional status" OR h*moglobin OR hb OR "maternal anemia" OR nutrient* OR "maternal health" OR "child health" OR malnourished OR "malarial anemia" OR plasmodi* OR falciparum OR malaria" anywhere and ""africa south of the sahara" OR "Sub-Saharan Africa" OR Subsahara OR "Central Africa" OR "middle Africa" OR "east africa" OR "Southern Africa" OR "west africa" OR Angola OR Benin OR Botswana OR "Burkina Faso" OR Burundi OR Cameroon OR "Cape Verde" OR "Central African Republic" OR Chad OR Comoros OR Congo OR "Cote d'Ivoire" OR Djibouti OR "Equatorial Guinea" OR Eritrea OR Ethiopia OR Gabon OR Gambia OR Ghana OR Guinea OR "Guinea-Bissau" OR Kenya OR Lesotho OR Liberia OR Madagascar OR Malawi OR Mali OR Mauritania OR Mauritius OR Mozambique OR Namibia OR Niger OR Nigeria OR Reunion OR Rwanda OR "Sao Tome and Principe" OR Senegal OR Seychelles OR "Sierra Leone" OR Somalia OR "South Africa" OR Sudan OR Swaziland OR Eswatini OR Tanzania OR Togo OR Uganda OR "Western Sahara" OR Zambia OR Zimbabwe"**

Filters: **Humans, English, Infant: birth-23 months, Infant: 1-23 months, Preschool Child: 2-5 years, from 2013 – 2024**

**Date of final search: April 30, 2024, Time coverage: Jan 1, 2013 – Apr 30, 2024**

# Appendix 3: List of extracted variables and Study Eligibility form

## List of extracted variables

1. Study report -
   1. Author (year)
   2. Language,
   3. title
2. Methods -
   1. Aim
   2. study type / design,
   3. country(s)
   4. setting
   5. sample size
   6. inclusion
   7. exclusion
3. Participants -
   1. mean age (SD)
   2. range,
   3. %female
4. Exposure(s) and Interventions –
   1. exposure(s)
   2. intervention(s)-detail,
   3. comparator(s)
5. Confounders –
   1. detail,% -
6. Outcomes -
   1. Health –
      1. outcome %/mean(sd)
      2. measure
   2. physical brain –
      1. construct
      2. %/mean(sd),
      3. Tool
      4. reliability/validity,
      5. deliberate validation (yes/no)
   3. Cognition –
      1. construct
      2. %/mean(sd)
      3. Tool
      4. reliability/validity
      5. deliberate validation (yes/no)
   4. developmental status/learning/disability –
      1. construct
      2. %/mean(sd)
      3. Tool
      4. reliability/validity
      5. deliberate validation (yes/no)
7. Timing of outcome assess
8. General findings
9. Outcome associations –
   1. Detail
   2. effects -

## Study eligibility form

**Brain growth, cognition and learning outcomes among early years children participating in anemia studies in sub-Saharan Africa: Protocol for a systematic review**

|  | **Eligibility factor – title and abstract** | **decision** | | | **Exclude reason** |
| --- | --- | --- | --- | --- | --- |
| **1** | **Type of Exposure / interventions** | | | | |
|  | Exposure is **anaemia,** i.e., defined as **iron deficiency in maternal or child**?  OR  Interventions of any form that prevent or treat anaemia:  E.g., maternal, infant, or child nutrition supplementation with any form of micronutrients including iron, vitamin A, etc., feeding programs, or others  Any **comparators** | Yes  Incl  ⇓ | Unclear  mark  ⇔ | No  Excl  ⇒ | 1 ineligible exposure or intervention |
| **2** | **Types of participants** | | | | |
|  | Children from **birth to 5 years**, i.e., **early years**, male and female  Children above 5 years are not eligible | Yes  Incl  ⇓ | Unclear  mark  ⇔ | No  Excl  ⇒ | 2 Ineligible participants |
| **3** | **Types of Outcomes** | | | | |
|  | Are outcomes measured any in the broad **neurodevelopment** and/or **learning** categories below?  Structural **brain growth**, **Cognition**, Learning ability, **child development status**, Brain activity, **Disability**, or Learning disorders.  If unsure, refer to descriptions in protocol. | Yes  Incl  ⇓ | Unclear  mark  ⇔ | No  Excl  ⇒ | 3 No eligible outcomes measured |
| **4** | **Timing** | | | | |
|  | Outcomes assessed at any one or more timepoints after birth and below 5 years? | Yes  Incl  ⇓ | Unclear  mark  ⇔ | No  Excl  ⇒ | 4 Ineligible timing of outcome assessments |
| **5** | **Location and/or setting** | | | | |
|  | studies conducted in **sub-Saharan Africa** region, any **country** in that region?  If unsure, refer to descriptions in protocol. | Yes  Incl  ⇓ | Unclear  mark  ⇔ | No  Excl  ⇒ | 5 Ineligible locations |
| **6** | **Report characteristics** | | | | |
|  | reports that are published from 2013 to 2024 and English | Yes  Incl  ⇓ | Unclear  mark  ⇔ | No  Excl  ⇒ | 6a before 2013  6b no English available |
| **7** | **Types of study** | | | | |
|  | quantitative, controlled trials, individually randomized RCTs or non-randomized, cluster trials, or before-after correlational study, with at least one data point after an intervention?  OR  cohort, cross-sectional, case series, and case report study that identify anaemia as risk factor of interest?  OR  Systematic reviews, meta-analysis, or other reports of anaemia exposure / intervention(s) and developmental outcomes | Yes  Incl  ⇓ | Unclear  mark  ⇔ | No  Excl  ⇒ | 7 Ineligible study type  Study / design does not position / report anaemia or anaemia-related disease among risks of interest |
|  | FINAL DECISION  Circle accordingly | INCLUDE  (7xYes) | UNCLEAR  (1xUnclear) | EXCLUDE  (1xNo) | |

All included will proceed to full text review for data extraction

# Appendix 4: List of studies excluded at full-text screening stage, with brief reasons.

| **Study** | **reasons** |
| --- | --- |
| 1. Adu-Afarwuah S. Impact of nutrient supplementation on maternal nutrition and child growth and development in sub-Saharan Africa: the case of small-quantity lipid-based nutrient supplements. *Matern Child Nutr* 2020; 16(suppl 3): e12960. DOI:10.1111/mcn.12960. | Narrative review; no or no relevant neurodevelopmental outcomes reported. |
| 1. Bangirana P, Datta D, Conroy AL, et al. Neurocognitive impairment in Ugandan children under 5 years of age with cerebral malaria or severe malarial anaemia occurs early and persists two years after illness. *Am J Trop Med Hyg* 2020; published online (ASTMH 2020 abstract). | Conference abstract only; full article not accessible at time of review. |
| 1. Maketa V, Mavoko HM, da Luz RI, et al. The relationship between Plasmodium infection, anaemia, and nutritional status in asymptomatic children aged under five years living in stable transmission zones in Kinshasa, Democratic Republic of Congo. *Malar J* 2015; 14: 83. DOI:10.1186/s12936-015-0595-5. | No or no relevant neurodevelopmental outcomes reported. |
| 1. Ewusie JE, Ahiadeke C, Beyene J, Hamid JS. Prevalence of anaemia among under-5 children in the Ghanaian population: estimates from the Ghana demographic and health survey. *BMC Public Health* 2014; 14: 626. DOI:10.1186/1471-2458-14-626. | No or no relevant neurodevelopmental outcomes reported. |
| 1. Baldi AJ, Larson LM, Pasricha SR. Balancing safety and potential for impact in universal iron interventions. *Nestle Nutr Inst Workshop Ser* 2020; 93: 51–62. DOI:10.1159/000503356. | No or no relevant neurodevelopmental outcomes reported. |
| 1. Tegegne M, Abate KH, Belachew T. Anaemia and associated factors among children aged 6–23 months in agrarian community of Bale zone: a cross-sectional study. *J Nutr Sci* 2022; 11: e96. DOI:10.1017/jns.2022.63. | No or no relevant neurodevelopmental outcomes reported. |
| 1. McCarthy EK, Kiely ME. The neonatal period: a missed opportunity for the prevention of iron deficiency and its associated neurological consequences? *Nutr Bull* 2019; 44: 309–19. DOI:10.1111/nbu.12407. | Narrative review; no or no relevant neurodevelopmental outcomes reported. |
| 1. Finkelstein JL, Fothergill A, Venkatramanan S, et al. Vitamin B12 supplementation during pregnancy for maternal and child health outcomes. *Cochrane Database Syst Rev* 2024; 1: CD013823. DOI:10.1002/14651858.CD013823.pub2. | Ineligible exposure/intervention; unclear outcomes |
| 1. Boivin MJ, Sikorskii A, Familiar-Lopez I, et al. Bouts of malaria illness as mediated by anaemia diminish cognitive development in very young Ugandan children. *Am J Trop Med Hyg* 2014; published online (ASTMH 2013 abstract). | Conference abstract only; duplicate of full article. |
| 1. Masuda K, Chitundu M. Multiple micronutrient supplementation using Spirulina platensis and infant growth, morbidity, and motor development: evidence from a randomised trial in Zambia. *PLoS One* 2019; 14: e0211693. DOI:10.1371/journal.pone.0211693. | Ineligible exposure/intervention (spirulina-based supplement; not anaemia/iron-focused). |
| 1. Boivin MJ, Sikorskii A, Familiar-Lopez I, et al. Malaria illness mediated by anaemia lessens cognitive development in younger Ugandan children. *Malar J* 2016; 15: 210. DOI:10.1186/s12936-016-1266-x. | Ineligible exposure/outcomes (malaria-mediated effects; not primary anaemia/iron focus). |
| 1. Gutata D. A case series: a mother and daughter with a critically low haemoglobin level resulting from severe anaemia secondary to malaria. *Int Med Case Rep J* 2024; 17: 149–55. DOI:10.2147/IMCRJ.S448712. | Case report; no or no relevant neurodevelopmental outcomes reported. |
| 1. Tesema GA, Worku MG, Tessema ZT, et al. Prevalence and determinants of severity levels of anaemia among children aged 6–59 months in sub-Saharan Africa: a multilevel ordinal logistic regression analysis. *PLoS One* 2021; 16: e0249978. DOI:10.1371/journal.pone.0249978. | No or no relevant neurodevelopmental outcomes reported. |
| 1. Bello-Manga H, DeBaun MR, Kassim AA. Epidemiology and treatment of relative anaemia in children with sickle cell disease in sub-Saharan Africa. *Expert Rev Hematol* 2016; 9: 1031–42. DOI:10.1080/17474086.2016.1240612. | Ineligible exposure/intervention (sickle cell disease); no relevant neurodevelopmental outcomes. |
| 1. Thejpal R. Iron deficiency in children. *S Afr Med J* 2015; 105: 607. DOI:10.7196/SAMJnew.7781. | No or no relevant neurodevelopmental outcomes reported. |
| 1. Bangirana P, Opoka RO, Boivin MJ, et al. Severe malarial anaemia is associated with long-term neurocognitive impairment. *Clin Infect Dis* 2014; 59: 336–44. DOI:10.1093/cid/ciu293. | Ineligible exposure/intervention (severe malarial anaemia; not primary anaemia/iron-deficiency question). |
| 1. Caulfield LE, Bose A, Chandyo RK, et al. Infant feeding practices, dietary adequacy, and micronutrient status measures in the MAL-ED study. *Clin Infect Dis* 2014; 59(suppl 4): S248–54. DOI:10.1093/cid/ciu421. | No or no relevant neurodevelopmental outcomes reported. |
| 1. Ocansey ME, Adu-Afarwuah S, Kumordzie SM, et al. The association of early linear growth and haemoglobin concentration with later cognitive, motor, and social-emotional development at preschool age in Ghana. *Matern Child Nutr* 2019; 15: e12834. DOI:10.1111/mcn.12834. | Ineligible population (older preschool only; outside 0–5y anaemia/IDA exposure definition for this review). |
| 1. Saye R, et al. Malaria chemoprevention, undernutrition and anaemia in children: findings from three randomised intervention trials in southern Mali. *Trop Med Int Health* 2015; 20(suppl): 1–147 (plenary abstr). DOI:10.1111/tmi.12575. | No or no relevant neurodevelopmental outcomes reported. |
| 1. Prado EL, Abbeddou S, Yakes Jimenez E, et al. Lipid-based nutrient supplements plus malaria and diarrhoea treatment increase infant development scores in a cluster-randomised trial in Burkina Faso. *J Nutr* 2016; 146: 814–22. DOI:10.3945/jn.115.225524. | Ineligible exposure/intervention (LNS plus infection treatment; not anaemia/iron-specific), considered background article. |
| 1. Prado EL, Abbeddou S, Jimenez EY, et al. Burkinabe infants given small-quantity lipid-based nutrient supplements and illness treatment in infancy score higher in motor, language, and personal-social development. *FASEB J* 2014; 28: 251·1 (abstr). DOI:10.1096/fasebj.28.1_supplement.251.1. | Conference abstract; background to included trial only. |
| 1. Beard JL, Hendricks MK, Perez EM, et al. Maternal iron deficiency anaemia affects postpartum emotions and cognition. *J Nutr* 2005; 135: 267–72. DOI:10.1093/jn/135.2.267. | Ineligible year (outside 2013–2024 search window). |
| 1. Gutema BT, Levecke B, Sorrie MB, et al. Effectiveness of intermittent iron and high-dose vitamin A supplementation on cognitive development of school children in southern Ethiopia: a randomised placebo-controlled trial. *Am J Clin Nutr* 2024; 119: 470–84. DOI:10.1016/j.ajcnut.2023.11.005. | Ineligible age group (school-aged children; outside 0–5 years). |
| 1. Fernandes M, Krebs N, Hambidge M, et al. Family care indices and linear growth predict INTER-NDA scores for child development at age 2 years: findings from the Women First trial. *Curr Dev Nutr* 2022; 6(suppl 1): 643. DOI:10.1093/cdn/nzac061.027. | Conducted outside sub-Saharan Africa. |
| 1. NCT00970866. Efficacy of lipid-based nutrient supplements for pregnant and lactating women and their infants. *ClinTrials.gov* 2009; NCT00970866 (trial registry record). | Ineligible year and no or no relevant neurodevelopmental outcomes reported. |
| 1. Christian AK, Atiglo DY, Okyere MA, Obeng-Dwamena A, Marquis GS, Jones AD. Women’s empowerment, children’s nutritional status, and the mediating role of household headship structure: evidence from sub-Saharan Africa. *Matern Child Nutr* 2023; 19: e13520. DOI:10.1111/mcn.13520. | No or no relevant neurodevelopmental outcomes reported. |
| 1. Ahmed F, Elnaiem W, Koko A, et al. Assessment of nutritional status of children with cerebral palsy and its association with degree of dysphagia and gross motor impairment. *Arch Dis Child* 2023; published online (RCPCH 2023 abstract). DOI:10.1136/archdischild-2023-rcpch.314. | Ineligible exposure/intervention (cerebral palsy/nutrition rehabilitation focus). |
| 1. Scarpa G, Berrang-Ford L, Galazoula M, et al. Identifying predictors for minimum dietary diversity and minimum meal frequency in children aged 6–23 months in Uganda. *Nutrients* 2022; 14: 5208. DOI:10.3390/nu14245208. | No or no relevant neurodevelopmental outcomes reported. |
| 1. Datta D, Bangirana P, Opoka RO, et al. Association of plasma tau with mortality and long-term neurocognitive impairment in survivors of paediatric cerebral malaria and severe malarial anaemia. *JAMA Netw Open* 2021; 4: e2138515. DOI:10.1001/jamanetworkopen.2021.38515. | Ineligible exposure/intervention (cerebral malaria and SMA; biomarker focus). |
| 1. Mburu W, Conroy AL, Cusick SE, et al. The impact of undernutrition on cognition in children with severe malaria and community children: a prospective 2-year cohort study. *J Trop Pediatr* 2021; 67: fma b091. DOI:10.1093/tropej/fmab091. | Ineligible exposure/intervention (severe malaria and undernutrition focus). |
| 1. Humphrey JH, Mbuya MNN, Ntozini R, et al. Independent and combined effects of improved water, sanitation, and hygiene, and improved complementary feeding, on child stunting and anaemia in rural Zimbabwe: a cluster-randomised trial. *Lancet Glob Health* 2019; 7: e132–47. DOI:10.1016/S2214-109X(18)30374-7. | Ineligible exposure/intervention and no or no relevant neurodevelopmental outcomes reported. |
| 1. Vray M, Hedible BG, Adam P, et al. A multicentre, randomised controlled comparison of three renutrition strategies for the management of moderate acute malnutrition among children aged 6–24 months (the MALINEA project). *Trials* 2018; 19: 3027. DOI:10.1186/s13063-018-3027-3. | Ineligible exposure/intervention (moderate acute malnutrition renutrition strategies). |
| 1. Ruisenor-Escudero H, Chandy J, Familiar Lopez I, et al. Association between plasma and cerebrospinal fluid biomarkers and neuropsychological outcomes among children with cerebral and severe malaria in Uganda. *Am J Trop Med Hyg* 2017; published online (ASTMH 2017 abstract). | Ineligible exposure/intervention (cerebral/severe malaria biomarker study). |
| 1. Lyatuu MB, Mkumbwa T, Stevenson R, et al. Planning and budgeting for nutrition programs in Tanzania: lessons learned from the national vitamin A supplementation program. *Int J Health Policy Manag* 2016; 5: 583–88. DOI:10.15171/ijhpm.2016.46. | Irrelevant to review question (programme planning/budgeting). |
| 1. Faber M, Laubscher R, Berti C. Poor dietary diversity and low nutrient density of the complementary diet for 6–24-month-old children in urban and rural KwaZulu-Natal, South Africa. *Matern Child Nutr* 2016; 12: 528–45. DOI:10.1111/mcn.12146. | Ineligible exposure/intervention (dietary diversity/complementary feeding; not anaemia/iron-focused). |
| 1. Liu E, Duggan C, Manji KP, et al. Multivitamin supplementation improves haematologic status in children born to HIV-positive women in Tanzania. *J Int AIDS Soc* 2013; 16: 180. DOI:10.7448/IAS.16.1.180. | No or no relevant neurodevelopmental outcomes reported. |
| 1. Masumo R, Bardsen A, Astrom AN. Developmental defects of enamel in primary teeth and association with early life-course events: a study of 6–36-month-old children in Manyara, Tanzania. *BMC Oral Health* 2013; 13: 21. DOI:10.1186/1472-6831-13-21. | Ineligible exposure/intervention (dental enamel defects; not anaemia/iron-focused neurodevelopment). |
| 1. Parpia T, Svensen E, Elwood S, et al. Cognitive outcomes at 18 months: findings from the Early Life Interventions for Childhood Growth and Development in Tanzania (ELICIT) trial. *Am J Trop Med Hyg* 2022; 106: 441–45. DOI:10.4269/ajtmh.21-0596. | Ineligible exposure/intervention (trial – infection/nutrition intervention; not anaemia/iron-specific). |
| 1. Custodio E, Herrador Z, Trigo E, et al. Nutrition supplementation plus standard of care versus standard of care alone or standard of care plus unconditional cash transfer in the prevention of chronic malnutrition in southern Angola: study protocol for the MuCCUA study, a cluster randomised controlled trial. *BMC Public Health* 2024; 24: 429. DOI:10.1186/s12889-024-17858-7. | Study protocol only; no or no relevant neurodevelopmental outcomes reported. |
| 1. Locks LM, Manji KP, McDonald CM, et al. The effect of daily zinc and/or multivitamin supplements on early childhood development in Tanzania: results from a randomised controlled trial. *Matern Child Nutr* 2017; 13: e12306. DOI:10.1111/mcn.12306. | Ineligible exposure/intervention (zinc/multivitamin; not anaemia/iron-focused). |
| 1. Osei Bonsu E, Addo IY, Boadi C, et al. Determinants of iron-rich food deficiency among children under 5 years in sub-Saharan Africa: a comprehensive analysis of Demographic and Health Surveys. *BMJ Open* 2024; 14: e079856. DOI:10.1136/bmjopen-2023-079856. | No or no relevant neurodevelopmental outcomes reported. |
| 1. Appiahene P, Dogbe SS, Kobina EE, et al. Application of ensemble models approach in anaemia detection using images of the palpable palm. *Med Novel Technol Devices* 2023; published online. | Irrelevant to review question (machine-learning anaemia detection from palm images). |
| 1. Ahmed M, Muhoozi GKM, Atukunda P, Westerberg AC, Iversen PO, Wangen KR. Cognitive development among children in a low-income setting: cost-effectiveness analysis of a maternal nutrition education intervention in rural Uganda. *PLoS One* 2023; 18: e0290379. DOI:10.1371/journal.pone.0290379. | Ineligible exposure/intervention (maternal nutrition education; not anaemia/iron-specific). |
| 1. Eshetu HB, Diress M, Belay DG, Seid MA, Chilot D, et al. Individual and community-level factors associated with iron-rich food consumption among children aged 6–23 months in Rwanda: a multilevel analysis of Rwanda Demographic and Health Survey. *PLoS One* 2023; 18: e0280466. DOI:10.1371/journal.pone.0280466. | Irrelevant to review question (iron-rich food consumption determinants only). |
| 1. Nkurunziza JC, Nabukeera-Barungi N, Kalyango JN, et al. Prevalence and factors associated with anaemia in children aged 6–24 months living in a high malaria transmission setting in Burundi. *PLoS One* 2022; 17: e0273651. DOI:10.1371/journal.pone.0273651. | No or no relevant neurodevelopmental outcomes reported. |
| 1. Aliyo A, Jibril A. Assessment of anaemia and associated risk factors among children under-five years old in the West Guji Zone, southern Ethiopia: hospital-based cross-sectional study. *PLoS One* 2022; 17: e0270853. DOI:10.1371/journal.pone.0270853. | No or no relevant neurodevelopmental outcomes reported. |
| 1. Golding J, Taylor C, Iles-Caven Y, Gregory S. The benefits of fish intake: results concerning prenatal mercury exposure and child outcomes from the ALSPAC prebirth cohort. *Neurotoxicology* 2022; 91: 22–30. DOI:10.1016/j.neuro.2022.04.012. | Irrelevant to review question (prenatal mercury exposure and fish intake; not anaemia/IDA). |
| 1. Wondemagegn AT, Mulu A. Effects of nutritional status on neurodevelopment of children aged under five years in East Gojjam, northwest Ethiopia, 2021: a community-based study. *Int J Gen Med* 2022; 15: 5533–45. DOI:10.2147/IJGM.S369408. | Ineligible exposure/intervention and outcomes (nutritional status; not anaemia/iron-focused exposure; broad neurodevelopment focus). |
| 1. Tilahun D, Yimer MA, Zamanuel TG. High magnitude of neonatal anaemia among sick newborns admitted to University of Gondar Comprehensive Specialized Hospital, northwest Ethiopia. *J Blood Med* 2022; 13: 293–302. DOI:10.2147/JBM.S361675. | No or no relevant neurodevelopmental outcomes reported. |
| 1. Orsango AZ, Loha E, Lindtjørn B, Engebretsen IMS. Co-morbid anaemia and stunting among children 2–5 years old in southern Ethiopia: a community-based cross-sectional study. *BMJ Paediatr Open* 2021; 5: e001039. DOI:10.1136/bmjpo-2021-001039. | No or no relevant neurodevelopmental outcomes reported. |
| 1. Owolabi AJ, Senbanjo IO, Oshikoya KA, et al. Multi-nutrient fortified dairy-based drink reduces anaemia without observed adverse effects on gut microbiota in anaemic malnourished Nigerian toddlers: a randomised dose–response study. *Nutrients* 2021; 13: 1566. DOI:10.3390/nu13051566. | No or no relevant neurodevelopmental outcomes reported. |
| 1. Field MS, Mithra P, Peña-Rosas JP. Wheat flour fortification with iron and other micronutrients for reducing anaemia and improving iron status in populations. *Cochrane Database Syst Rev* 2021; 1: CD011302. DOI:10.1002/14651858.CD011302.pub3. | Conducted outside sub-Saharan Africa (global review of flour fortification). |
| 1. Kancherla V, Chadha M, Rowe L, et al. Reducing the burden of anaemia and neural tube defects in low-income and middle-income countries: an analysis to identify countries with an immediate potential to benefit from large-scale mandatory fortification of wheat flour and rice. *Nutrients* 2021; 13: 244. DOI:10.3390/nu13010244. | Irrelevant to review question (fortification policy modelling; not child neurodevelopment). |
| 1. Markova V, Holm C, Pinborg AB, Thomsen LL, Moos T. Impairment of the developing human brain in iron deficiency: correlations to findings in experimental animals and prospects for early intervention therapy. *Pharmaceuticals* 2019; 12: 120. DOI:10.3390/ph12030120. | Narrative review; used for background on iron deficiency and brain development. |
| 1. Alemayehu M, Meskele M, Alemayehu B, Yakob B. Prevalence and correlates of anaemia among children aged 6–23 months in Wolaita zone, southern Ethiopia. *PLoS One* 2019; 14: e0206268. DOI:10.1371/journal.pone.0206268. | Ineligible population and no or no relevant neurodevelopmental outcomes reported. |
| 1. Finkelstein JL, Fothergill A, Hackl LS, Haas JD, Mehta S. Iron biofortification interventions to improve iron status and functional outcomes. *Proc Nutr Soc* 2019; 78: 197–207. DOI:10.1017/S0029665118002847. | Ineligible population (global biofortification review; not SSA child neurodevelopment). |
| 1. Engidaye G, Melku M, Yalew A, et al. Undernutrition, maternal anaemia, and household food insecurity are risk factors of anaemia among pre-school aged children in Menz Gera Midir district, eastern Amhara, Ethiopia: a community-based cross-sectional study. *BMC Public Health* 2019; 19: 968. DOI:10.1186/s12889-019-7293-0. | Ineligible population and no or no relevant neurodevelopmental outcomes reported. |
| 1. Melku M, Takele WW, Anlay DZ, et al. Male and undernourished children were at high risk of anaemia in Ethiopia: a systematic review and meta-analysis. *Ital J Pediatr* 2018; 44: 79. DOI:10.1186/s13052-018-0513-x. | No or no relevant neurodevelopmental outcomes reported. |
| 1. Getaneh Z, Enawgaw B, Engidaye G, et al. Prevalence of anaemia and associated factors among school children in Gondar town public primary schools, northwest Ethiopia: a school-based cross-sectional study. *PLoS One* 2017; 12: e0190151. DOI:10.1371/journal.pone.0190151. | Ineligible population (school-age children). |
| 1. Misganaw A, Melaku YA, Tessema GA, et al. National disability-adjusted life years (DALYs) for 257 diseases and injuries in Ethiopia, 1990–2015: findings from the Global Burden of Disease Study 2015. *Popul Health Metr* 2017; 15: 28. DOI:10.1186/s12963-017-0146-0. | Irrelevant to review question (national DALYs overview). |
| 1. Stecher CW, Sacko M, Madsen H, et al. Anaemia and growth retardation associated with Schistosoma haematobium infection in Mali: a possible subtle impact of a neglected tropical disease. *Trans R Soc Trop Med Hyg* 2017; 111: 144–53. DOI:10.1093/trstmh/trx037. | Ineligible exposure/intervention (schistosomiasis-related anaemia). |
| 1. Brown KH, Engle-Stone R, Kagin J, Rettig E, Vosti SA. Use of optimisation modelling for selecting national micronutrient intervention strategies: an example based on potential programs for control of vitamin A deficiency in Cameroon. *Food Nutr Bull* 2015; 36(suppl 3): S141–8. DOI:10.1177/0379572115599325. | Irrelevant to review question (vitamin A intervention modelling). |
| 1. Chikhungu LC, Madise NJ. Seasonal variation of child undernutrition in Malawi: is seasonal food availability an important factor? Findings from a national level cross-sectional study. *BMC Public Health* 2014; 14: 1146. DOI:10.1186/1471-2458-14-1146. | Irrelevant to review question (seasonal undernutrition; not anaemia/IDA exposure with neurodevelopment). |
| 1. Owa OT, Brown BJ, Adeodu OO. Iron deficiency among apparently healthy children aged 6–24 months in Ibadan, Nigeria. *Pediatr Hematol Oncol* 2016; 33: 338–46. DOI:10.1080/08880018.2016.1217110. | No or no relevant neurodevelopmental outcomes reported. |
| 1. Hickson MR, Conroy AL, Bangirana P, et al. Acute kidney injury in Ugandan children with severe malaria is associated with long-term behavioural problems. *PLoS One* 2019; 14: e0226405. DOI:10.1371/journal.pone.0226405. | Ineligible exposure/intervention (severe malaria and AKI; behavioural outcomes not in anaemia/IDA context). |
| 1. Villar J, Fernandes M, Purwar M, et al. Neurodevelopmental milestones and associated behaviours are similar among healthy children across diverse geographical locations. *Nat Commun* 2019; 10: 511. DOI:10.1038/s41467-018-07983-4. | Conducted outside sub-Saharan Africa (multi-country; not SSA-specific). |
| 1. Winje BA, Kvestad I, Krishnamachari S, et al. Does early vitamin B12 supplementation improve neurodevelopment and cognitive function in childhood and into school age: a study protocol for extended follow-ups from randomised controlled trials in India and Tanzania. *BMJ Open* 2018; 8: e018962. DOI:10.1136/bmjopen-2017-018962. | Ineligible exposure/intervention; no or no relevant child neurodevelopment outcomes available at review time. |
| 1. Tiku YS, Mekonnen TC, Workie SB, Amare E. Does anaemia have major public health importance in children aged 6–59 months in the Duggina Fanigo district of Wolaita zone, southern Ethiopia? *Ann Nutr Metab* 2018; 72: 3–11. DOI:10.1159/000484324. | No or no relevant neurodevelopmental outcomes reported. |
| 1. Melku M, Alene KA, Terefe B, et al. Anaemia severity among children aged 6–59 months in Gondar town, Ethiopia: a community-based cross-sectional study. *Ital J Pediatr* 2018; 44: 107. DOI:10.1186/s13052-018-0547-0. | No or no relevant neurodevelopmental outcomes reported. |
| 1. Fernald LC, Galasso E, Qamruddin J, et al. A cluster-randomised, controlled trial of nutritional supplementation and promotion of responsive parenting in Madagascar: the MAHAY study design and rationale. *BMC Public Health* 2016; 16: 466. DOI:10.1186/s12889-016-3097-7. | No or no relevant neurodevelopmental outcomes reported (study design rationale only). |
| 1. Halliday D, Lloyd-Fox S, Begus K, et al. Brain imaging of nutrition-related cognitive development in rural Gambia: studies from birth to 24 months of age. *FASEB J* 2014; 28: 619·1 (abstr). DOI:10.1096/fasebj.28.1_supplement.619.1. | Conference abstract only; insufficient neuroimaging and developmental detail. |
| 1. Oluwole OB, Noll RB, Winger DG, Akinyanju O, Novelli EM. Cognitive functioning in children from Nigeria with sickle cell anaemia. *Pediatr Blood Cancer* 2016; 63: 1990–97. DOI:10.1002/pbc.26126. | Ineligible exposure/intervention (sickle cell anaemia). |
| 1. Blum LS, Swartz H, Olisenekwu G, Erhabor I, Gonzalez W. Social and economic factors influencing intrahousehold food allocation and egg consumption of children in Kaduna State, Nigeria. *Matern Child Nutr* 2023; 19: e13442. DOI:10.1111/mcn.13442. | Irrelevant to review question (food allocation/egg consumption; no neurodevelopmental outcomes). |
| 1. Larson LM, Kubes JN, Ramírez-Luzuriaga MJ, Khishen S, Shankar AH, Prado EL. Effects of increased haemoglobin on child growth, development, and disease: a systematic review and meta-analysis. *Ann N Y Acad Sci* 2019; 1450: 83–104. DOI:10.1111/nyas.14105. | Conducted outside sub-Saharan Africa (global systematic review/meta-analysis). |
| 1. Mhanna RG, Rahal M, Iskandarani M, Hammoudi D. Iron deficiency anaemia in Lebanese infants. *Int J Pharm Pract* 2016; 24: 203–8. DOI:10.1111/ijpp.12236. | No or no relevant neurodevelopmental outcomes reported. |
| 1. Lutter CK, Iannotti LL, Stewart CP. The potential of a simple egg to improve maternal and child nutrition. *Matern Child Nutr* 2018; 14(suppl 3): e12678. DOI:10.1111/mcn.12678. | Irrelevant to review question (general nutrition commentary on eggs; no anaemia/IDA-linked neurodevelopment outcomes). |

# Appendix 5. PRISMA Checklist^[[1]](#footnote-1)^

| **Section and Topic** | **Item #** | **Checklist item** | **Location where item is reported, pg#** |
| --- | --- | --- | --- |
| **TITLE** | | |  |
| Title | 1 | Identify the report as a systematic review. | 1,2 |
| **ABSTRACT** | | |  |
| Abstract | 2 | See the PRISMA 2020 for Abstracts checklist. | 2 |
| **INTRODUCTION** | | |  |
| Rationale | 3 | Describe the rationale for the review in the context of existing knowledge. | 3 |
| Objectives | 4 | Provide an explicit statement of the objective(s) or question(s) the review addresses. | 3 |
| **METHODS** | | |  |
| Eligibility criteria | 5 | Specify the inclusion and exclusion criteria for the review and how studies were grouped for the syntheses. | 4,5, search strategy and selection criteria |
| Information sources | 6 | Specify all databases, registers, websites, organisations, reference lists and other sources searched or consulted to identify studies. Specify the date when each source was last searched or consulted. | 4 |
| Search strategy | 7 | Present the full search strategies for all databases, registers and websites, including any filters and limits used. | 4, Appendix 2, |
| Selection process | 8 | Specify the methods used to decide whether a study met the inclusion criteria of the review, including how many reviewers screened each record and each report retrieved, whether they worked independently, and if applicable, details of automation tools used in the process. | 4 |
| Data collection process | 9 | Specify the methods used to collect data from reports, including how many reviewers collected data from each report, whether they worked independently, any processes for obtaining or confirming data from study investigators, and if applicable, details of automation tools used in the process. | 4 |
| Data items | 10a | List and define all outcomes for which data were sought. Specify whether all results that were compatible with each outcome domain in each study were sought (e.g. for all measures, time points, analyses), and if not, the methods used to decide which results to collect. | 4, appendix 3, data extraction |
|  | 10b | List and define all other variables for which data were sought (e.g. participant and intervention characteristics, funding sources). Describe any assumptions made about any missing or unclear information. | 4, appendix 3, results, discussion |
| Study risk of bias assessment | 11 | Specify the methods used to assess risk of bias in the included studies, including details of the tool(s) used, how many reviewers assessed each study and whether they worked independently, and if applicable, details of automation tools used in the process. | 5, 7, appendix 4 |
| Effect measures | 12 | Specify for each outcome the effect measure(s) (e.g. risk ratio, mean difference) used in the synthesis or presentation of results. | 4, 5-7 |
| Synthesis methods | 13a | Describe the processes used to decide which studies were eligible for each synthesis (e.g. tabulating the study intervention characteristics and comparing against the planned groups for each synthesis (item #5)). | 4, table 1 |
|  | 13b | Describe any methods required to prepare the data for presentation or synthesis, such as handling of missing summary statistics, or data conversions. | 4, data synthesis |
|  | 13c | Describe any methods used to tabulate or visually display results of individual studies and syntheses. | 5-7, tables 1,2,3 |
|  | 13d | Describe any methods used to synthesize results and provide a rationale for the choice(s). If meta-analysis was performed, describe the model(s), method(s) to identify the presence and extent of statistical heterogeneity, and software package(s) used. | 5,6 |
|  | 13e | Describe any methods used to explore possible causes of heterogeneity among study results (e.g. subgroup analysis, meta-regression). | n/a |
|  | 13f | Describe any sensitivity analyses conducted to assess robustness of the synthesized results. | n/a |
| Reporting bias assessment | 14 | Describe any methods used to assess risk of bias due to missing results in a synthesis (arising from reporting biases). | 7, appendix 4 |
| Certainty assessment | 15 | Describe any methods used to assess certainty (or confidence) in the body of evidence for an outcome. | n/a |
| **RESULTS** | | |  |
| Study selection | 16a | Describe the results of the search and selection process, from the number of records identified in the search to the number of studies included in the review, ideally using a flow diagram. | 5, Figure 1 |
|  | 16b | Cite studies that might appear to meet the inclusion criteria, but which were excluded, and explain why they were excluded. | 5, appendix 5 |
| Study characteristics | 17 | Cite each included study and present its characteristics. | 5, table 1 |
| Risk of bias in studies | 18 | Present assessments of risk of bias for each included study. | 7, appendix 4 |
| Results of individual studies | 19 | For all outcomes, present, for each study: (a) summary statistics for each group (where appropriate) and (b) an effect estimate and its precision (e.g. confidence/credible interval), ideally using structured tables or plots. | Tables 1, 2,3 |
| Results of syntheses | 20a | For each synthesis, briefly summarise the characteristics and risk of bias among contributing studies. | 5-7 |
|  | 20b | Present results of all statistical syntheses conducted. If meta-analysis was done, present for each the summary estimate and its precision (e.g. confidence/credible interval) and measures of statistical heterogeneity. If comparing groups, describe the direction of the effect. | n/a |
|  | 20c | Present results of all investigations of possible causes of heterogeneity among study results. | discussion |
|  | 20d | Present results of all sensitivity analyses conducted to assess the robustness of the synthesized results. | n/a |
| Reporting biases | 21 | Present assessments of risk of bias due to missing results (arising from reporting biases) for each synthesis assessed. | 7, discussion |
| Certainty of evidence | 22 | Present assessments of certainty (or confidence) in the body of evidence for each outcome assessed. | 7 |
| **DISCUSSION** | | |  |
| Discussion | 23a | Provide a general interpretation of the results in the context of other evidence. | 7,8 |
|  | 23b | Discuss any limitations of the evidence included in the review. | 8 |
|  | 23c | Discuss any limitations of the review processes used. | 8 |
|  | 23d | Discuss implications of the results for practice, policy, and future research. | 9 |
| **OTHER INFORMATION** | | |  |
| Registration and protocol | 24a | Provide registration information for the review, including register name and registration number, or state that the review was not registered. | 1,2, abstract |
|  | 24b | Indicate where the review protocol can be accessed, or state that a protocol was not prepared. | 1,2, abstract |
|  | 24c | Describe and explain any amendments to information provided at registration or in the protocol. | n/a |
| Support | 25 | Describe sources of financial or non-financial support for the review, and the role of the funders or sponsors in the review. | 2, abstract, 5, 9, |
| Competing interests | 26 | Declare any competing interests of review authors. | 9 |
| Availability of data, code and other materials | 27 | Report which of the following are publicly available and where they can be found: template data collection forms; data extracted from included studies; data used for all analyses; analytic code; any other materials used in the review. | 9 |

1. *From:*  Page MJ, McKenzie JE, Bossuyt PM, Boutron I, Hoffmann TC, Mulrow CD, et al. The PRISMA 2020 statement: an updated guideline for reporting systematic reviews. BMJ 2021;372:n71. doi: 10.1136/bmj.n71

   For more information, visit: <http://www.prisma-statement.org/> [↑](#footnote-ref-1)
